# Approaching a “Naked” Boryl Anion: Amide Metathesis as a Route to Calcium, Strontium, and Potassium Boryl Complexes

**DOI:** 10.1002/anie.202011839

**Published:** 2020-11-23

**Authors:** Andrey V. Protchenko, Petra Vasko, M. Ángeles Fuentes, Jamie Hicks, Dragoslav Vidovic, Simon Aldridge

**Affiliations:** ^1^ Inorganic Chemistry Laboratory Department of Chemistry University of Oxford South Parks Road Oxford OX1 3QR UK; ^2^ Department of Chemistry, Nanoscience Center University of Jyväskylä P. O. Box 35 40014 Jyväskylä Finland

**Keywords:** atoms in molecules, boron, boryl, s-block chemistry, structural studies

## Abstract

Amide metathesis has been used to generate the first structurally characterized boryl complexes of calcium and strontium, {(Me_3_Si)_2_N}M{B(NDippCH)_2_}(thf)_*n*_ (M=Ca, *n*=2; M=Sr, *n*=3), through the reactions of the corresponding bis(amides), M{N(SiMe_3_)_2_}_2_(thf)_2_, with (thf)_2_Li‐ {B(NDippCH)_2_}. Most notably, this approach can also be applied to the analogous potassium amide K{N(SiMe_3_)_2_}, leading to the formation of the solvent‐free borylpotassium dimer [K{B(NDippCH)_2_}]_2_, which is stable in the solid state at room temperature for extended periods (48 h). A dimeric structure has been determined crystallographically in which the K^+^ cations interact weakly with both the ipso‐carbons of the flanking Dipp groups and the boron centres of the diazaborolyl heterocycles, with K⋅⋅⋅B distances of >3.1 Å. These structural features, together with atoms in molecules (QTAIM) calculations imply that the boron‐containing fragment closely approaches a limiting description as a “free” boryl anion in the condensed phase.

## Graphical abstract

Amide metathesis is exploited as a route to boryl compounds of K, Ca, and Sr. The dimeric potassium system is shown by crystallographic and atoms‐in‐molecules studies to approach a limiting description as a “free” boryl anion.

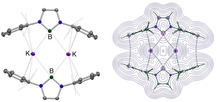

Boryl (or “boranyl”) anions, [BX_2_]^−^, featuring an electronic sextet at boron,[^1^, ^2^] were long targeted as synthetic reagents offering the possibility for “umpolung” nucleophilic reactivity at boron. Such systems are isoelectronic with charge neutral carbenes, CX_2_,^[3]^ and the molecular design features which led to the isolation of the first anionic boryl nucleophiles resemble closely those employed for the stabilization of N‐heterocyclic carbenes (i.e. containment within a five‐membered heterocycle, use of electronegative but π‐donating α‐N substituents and high steric loading in the vicinity of the boron/carbon centre).[^4^, ^5^]

In terms of the “partner” metal, boryllithium compounds had been advanced—on the basis of quantum chemical calculations—as being attractive synthetic targets even before their experimental realization.^[6]^ In part, this reflects enhanced covalency (compared, for example, to heavier group 1 metal derivatives), but also the stabilization of the singlet ground state (relative to the triplet state) in the presence of a metal cation at relatively short M‐B distances.^[6a]^ In the absence of such a cation, the energetic separation between these two states is calculated to be very small.^[6]^ As such, it is unsurprising that the only examples of structurally characterized boryl compounds from among the s‐block metals reported to date feature lithium from group 1 (e.g. **I**–**V**, Figure 1),[^4^, ^7^] or either beryllium or magnesium from group 2 (**VI**–**IX**).[^8^, ^9^] Such observations are consistent with enhanced covalent character and the reduced lability associated with (for example) alkyl derivatives of these metals compared to their heavier congeners. More ionic boryl systems analogous to recently reported potassium aluminyl compounds (e.g. **X**) are, to our knowledge, unknown.^[10]^

**Figure 1:**
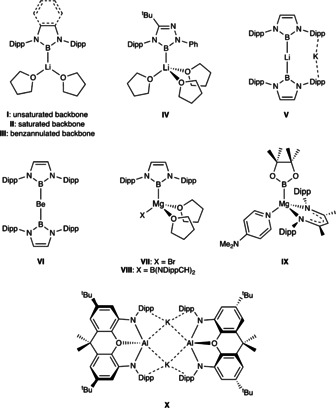
Key previously reported examples of s‐block boryl (and related) complexes of relevance to this study. (Dipp=2,6‐di*iso*propylphenyl).

Metathesis reactions of group 2 metal amides have been widely employed in the formation of M−E bonds (e.g. in the synthesis of solvent‐free dimethylcalcium by Anwander et al.),^[11]^ and have been elegantly exploited in catalytic processes leading to a range of hydro‐elementation reactions.^[12]^ Hill and co‐workers have also exploited a similar exchange process in the formation of Mg−B bonds from a Mg−C bonded precursor and B_2_pin_2_ (pin=OCMe_2_CMe_2_O),^[9b]^ With this in mind, we perceived that the reactions of amide derivatives of the heavier group 2 elements such as Ca{N(SiMe_3_)_2_}_2_(thf)_2_ and Sr{N(SiMe_3_)_2_}_2_(thf)_2_ with Yamashita's boryllithium reagent (thf)_2_Li{B(NDippCH)_2_} (**I**, where Dipp=2,6‐di*iso*propylphenyl)[^7a^, ^7b^] might provide access to hitherto unreported examples of group 2 metal‐boron bonds. Moreover, given the ready availability of the thf‐free potassium amide K{N(SiMe_3_)_2_}, we also hypothesized that analogous chemistry might permit access to a donor‐free borylpotassium species. We envisaged that such a compound would be of interest not only from a structural perspective (given the dimeric, predominantly ionic structures determined recently for related potassium aluminyl systems of the type [K(AlX_2_)]_2_),^[10]^ but also as an alternative lithium‐free source of the boryl anion of use, for example, in the formation of *f*‐element or early *d*‐block complexes.[^13^, ^14^]

The reactions of M{N(SiMe_3_)_2_}_2_(thf)_2_ (M=Ca, Sr) with one equivalent of (thf)_2_Li{B(NDippCH)_2_} in diethyl ether generate the corresponding heteroleptic amido boryl complexes {(Me_3_Si)_2_N}M{B(NDippCH)_2_}(thf)_*n*_ (**1**: M=Ca, *n*=2; **2**: M=Sr, *n*=3), which can be isolated as crystalline solids in 40–50 % yield (Scheme 1). These compounds are labile in benzene solution at room temperature (particularly for M=Sr), but the structures of both systems in the solid state can be determined by X‐ray crystallography.

**Scheme 1:**
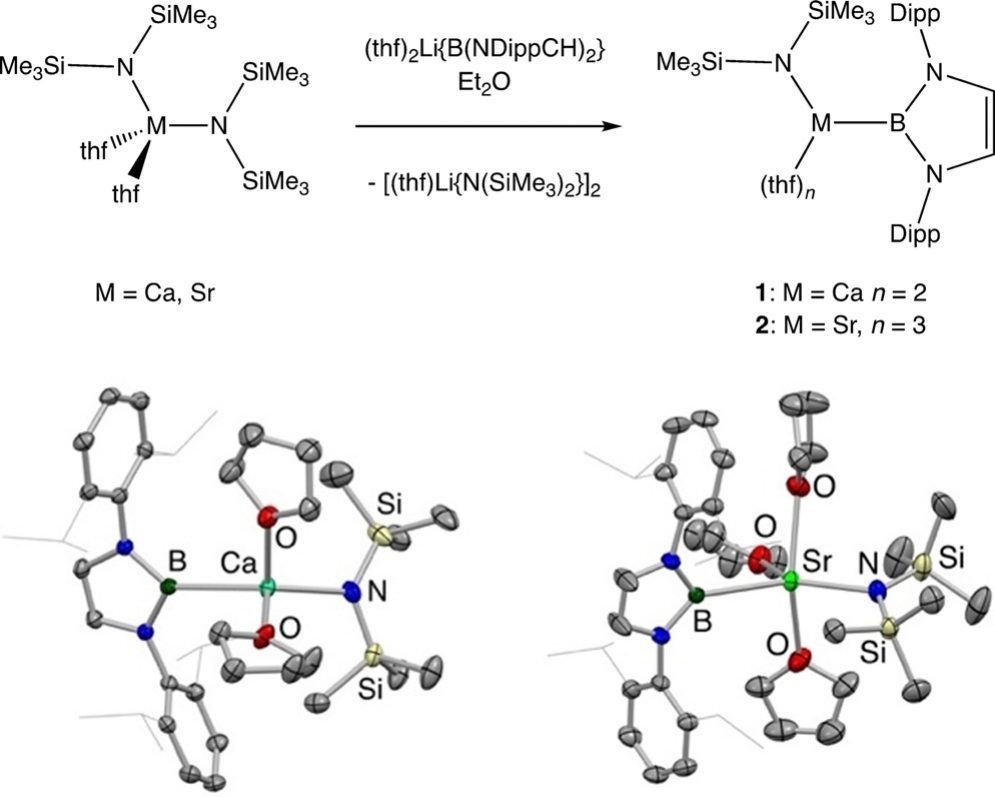
(top) Syntheses of {(Me_3_Si)_2_N}Ca{B(NDippCH)_2_}(thf)_2_ (**1**) and {(Me_3_Si)_2_N}Sr{B(NDippCH)_2_}(thf)_3_ (**2**), from the corresponding metal bis(amide) via metathesis with boryllithium (Dipp=2,6‐^*i*^Pr_2_C_6_H_3_). (bottom) Molecular structures of **1** (left) and **2** (right) in the solid state as determined by X‐ray crystallography. Second component of the asymmetric unit (for **1**) and hydrogen atoms omitted, and isopropyl groups shown in wireframe format for clarity. Thermal ellipsoids set at the 35 % probability level. Key distances [Å]: (for **1**) Ca–B 2.704(4), Ca–N 2.323(3), Ca–O 2.374(2), 2.395(2); (for **2**) Sr–B 2.984(3), Sr–N 2.505(3), Sr–O 2.505(3), 2.587(3), 2.565(13).

While boryl complexes of beryllium^[7]^ and magnesium^[8]^ have been reported previously,^[15]^ **1** and **2** represent to our knowledge the first examples of related systems featuring the heavier group 2 elements. Attempts to synthesize the corresponding barium compound, however, were unsuccessful, with the boranes HB(NDippCH)_2_ and PhB(NDippCH)_2_ being found to be the main components of a reaction mixture derived from Ba{N(SiMe_3_)_2_}_2_(thf)_3_ and (thf)_2_Li‐ {B(NDippCH)_2_} in benzene.^[16]^

In the solid state, the structures of **1** and **2** are superficially similar, differing primarily in the number of thf molecules retained within the coordination sphere of the respective metal centres. This increases from two (Ca) to three (Sr), and is presumably a contributory factor in the finding that the M‐B distances increase more markedly going down group 2 than would be expected purely on the basis of simple covalent radius arguments [*d*(M−B)=2.704(4) (Ca), 2.984(3) Å (Sr), *cf. r*_cov_=1.76 (Ca), 1.95 Å (Sr), respectively]. The M‐B distances for both **1** and **2** actually fall outside of the sum of the respective covalent radii [*r*_cov_(B)=0.84 Å],^[17]^ consistent with the idea of a predominantly electrostatic interaction between the boryl ligand and the metal centre. By contrast, the Ca‐N and Sr‐N distances for the same two complexes [2.323(3) and 2.505(3) Å] fall well *within* the sum of the respective covalent radii [*r*_cov_(N)=0.71 Å].^[17]^

Given the charge‐separated nature of the Ca and Sr boryl systems implied by these structural results, we wondered whether the boryl/amide metathesis approach could be extended to potassium amides K[NR_2_]. In this case the absence of an additional co‐ligand might allow access to an effectively “ionic salt” of the type M^+^[boryl]^−^. Accordingly, combination of (thf)_2_Li{B(NDippCH)_2_}, K{N(SiMe_3_)_2_} and hexane at 77 K, followed by warming to room temperature, and crystallization from the same solvent at 243 K leads to the formation of deep yellow single crystals in ca. 50 % yield. This product can be shown by X‐ray crystallography to be the solvent‐free centrosymmetric borylpotassium dimer, **3** (Scheme 2), which is stable in the solid state at room temperature for 2 days under an inert atmosphere.

**Scheme 2:**
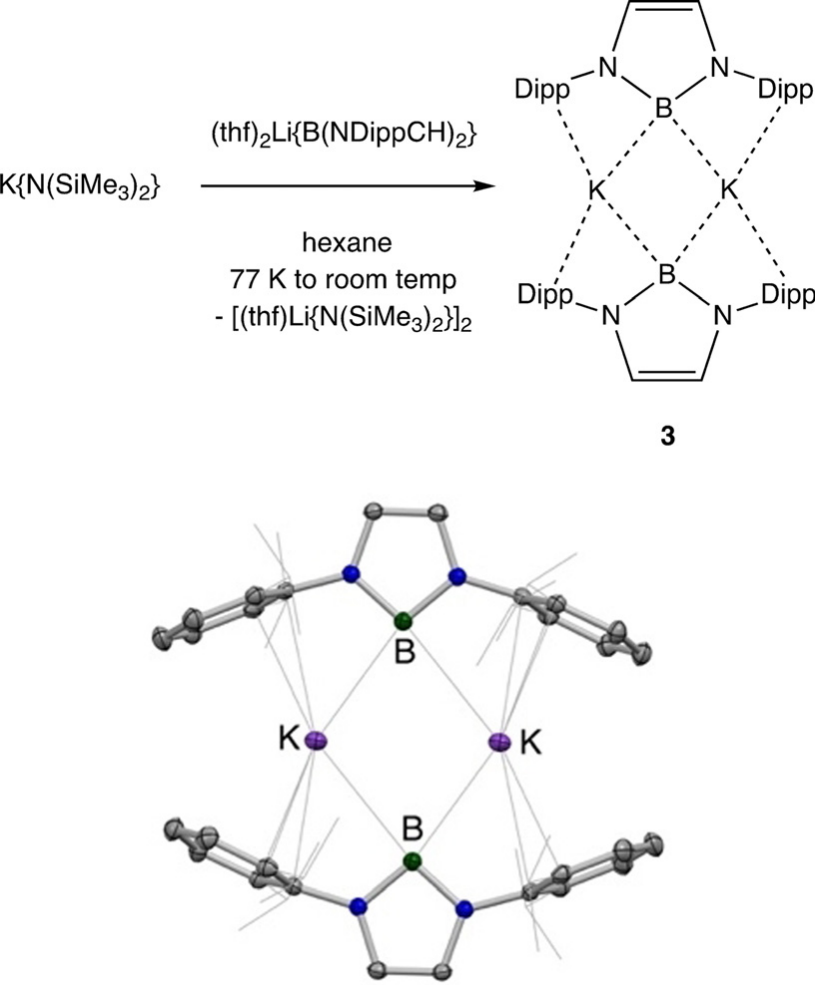
(top) Synthesis of borylpotassium dimer [K{B(NDippCH)_2_}]_2_ (**3**) using an amide metathesis approach. (bottom) Molecular structure of **3** in the solid state as determined by X‐ray crystallography. Hydrogen atoms omitted, and isopropyl groups shown in wireframe format for clarity. Thermal ellipsoids set at the 35 % probability level. Key distances [Å] and angles [°]: N–B 1.472(2), 1.473(2), K–B 3.105(2), 3.244(2), K–C_ipso_ 3.096(2), 3.201(2); N‐B‐N 98.2(1).

The overarching structural features of **3** in the solid state resemble those reported previously for related potassium aluminyl systems (e.g. **X**),^[10]^ featuring a dimeric structure in which two formally anionic diazaboryl units are held together by a pair of K^+^ counter‐ions. These cations engage in contacts with the arene rings of the flanking Dipp groups in a manner similar to that observed for the K^+^ ion in **V**;^[7e]^ the closest K⋅⋅⋅C separation [3.096(2) Å] involves the *ipso*‐carbon of each Dipp substituent. A similar distance defines the closest K⋅⋅⋅B separation [3.105(2) Å], which is therefore well outside the sum of the respective covalent radii (2.03 + 0.84=2.87 Å).^[17]^ The other K ⋅⋅B contact associated with each of the boron centres in the solid state is even longer [3.244(2) Å], with the positioning of the potassium centres either side of the B⋅⋅⋅B axis also suggesting relatively poor orbital overlap with the boron‐based lone pairs (Figure 2). Atoms in Molecules (QTAIM) calculations were carried out on the model system **3′**, in which the ^*i*^Pr substituents in the 2‐/6‐positions of the aryl groups of dimeric **3** were replaced by Me groups for computational efficiency. This analysis reveals the presence of a bond path between each of the K and B centres in **3′** (Figure 2), and also between the K centres and the *ipso* carbons of the flanking rings. However, the electron density at the bond critical points, *ρ*(*r*), corresponding to the K−B bond paths is very low (0.0121 e Å^−3^). By means of comparison, the corresponding electron density calculated at the Li−B bond critical point in **I** (using an identical method) is 0.0297 e Å^−3^, consistent with the idea that the metal‐boron interaction in **3** is significantly more ionic in nature.

**Figure 2:**
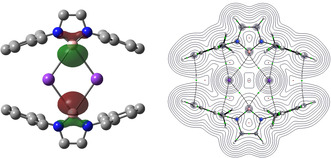
(left) HOMO (−3.80 eV, −367 kJ mol^−1^) for **3′** as calculated by DFT (PBE1PBE, Def‐TZVP, GD3BJ); the LUMO (at −0.38 eV/ −37 kJ mol^−1^) is centred on the flanking Dipp rings. (right) QTAIM analysis of **3′** showing bond critical points (BCPs) in green. The values of *ρ*(*r*) and ∇^2^ *ρ*(*r*) at the BCPs between K and B are 0.0121 e Å^−3^ and 0.0327 e Å^−5^, respectively.

**3** is the first example of lithium‐free group 1 boryl complex, and based on its solid‐state structure appears to offer the closest approach yet to the idea of a “free” boryl anion in the condensed phases. The M⋅⋅⋅B distances in **3** are >0.23 Å longer than the sum of the respective covalent radii (cf. 0.1–0.15 Å for boryllithium derivatives),^[17]^ and the geometric features of the diazaborolyl heterocycle itself are in closest agreement with those calculated for the “free” anion. Notably the N‐B‐N angle (which is known to be sensitive to the nature of a covalent B‐bound substituent)^[6]^ is very close to the value calculated for the gas‐phase anion [98.2(1) vs. 97.8°].^[7a]^ The energy gap between (fully optimized) singlet and triplet states for the model system **3′** is calculated to be 166 kJ mol^−1^.

Consistent with the predominantly ionic nature of **3**, it proves to be extremely reactive in solution: in benzene it reacts completely over a period of ca. 60 min at room temperature. Interestingly, the reaction pathway is distinct from that observed for calcium/strontium boryl complexes **1** and **2**, and for the potassium diboryllithiate system **V** reported by Yamashita (each of which generates a mixture of HB(NDippCH)_2_ and PhB(NDippCH)_2_ by activation of the benzene solvent).[^7e^, ^18^] **3**, by contrast, predominantly undergoes an *intra*molecular C−H activation process via formal oxidative addition at boron of a methine C−H bond of one of the flanking Dipp groups (to give **4**, Scheme 3).^[19]^ A similar mode of reactivity has been postulated for the transient boryllithium complex generated from BrB(NMesCH_2_)_2_ and lithium metal, with both deprotonation and H‐atom transfer (radical) mechanisms having been proposed for the formation of the B−H and B−C bonds.^[7a]^ In the case of **3**, the dimeric nature of the precursor is retained in the C−H activation product **4**, with additional K⋅⋅⋅C contacts in the solid state linking these units further into a loosely‐bound one‐dimensional coordination polymer.

**Scheme 3:**
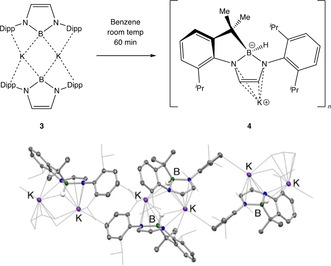
(top) Intramolecular C−H activation of a Dipp methine group in **3** to give **4** at room temperature in arene solvents. (bottom) Molecular structure of **4** in the solid state as determined by X‐ray crystallography. Hydrogen atoms omitted, and isopropyl groups shown in wireframe format for clarity. Thermal ellipsoids set at the 35 % probability level. Key distances [Å] and angles [°]: N–B 1.576(3), 1.584(2), C–B 1.663(3), B–H 1.14(2); N‐B‐N 100.1(1).

In summary, we report on a conceptually simple metathesis approach for the generation of highly polar boryl derivatives of potassium, calcium and strontium from a boryllithium precursor and bis(trimethylsilyl)amide derivatives of the respective metals. In the case of the potassium boryl product, a dimeric solvent‐free structure is observed in the solid state in which the K^+^ cations interact weakly with both the ipso‐carbons of the flanking Dipp groups and the boron centres of the diazaborolyl heterocycles. These structural features, together with QTAIM calculations imply that the boron‐containing fragment closely approaches a limiting description as a “free” boryl anion in the condensed phase.^[20]^

## Conflict of interest

The authors declare no conflict of interest.

## Supporting information

As a service to our authors and readers, this journal provides supporting information supplied by the authors. Such materials are peer reviewed and may be re‐organized for online delivery, but are not copy‐edited or typeset. Technical support issues arising from supporting information (other than missing files) should be addressed to the authors.

Supplementary

Supplementary
