# Supplementary material for: Approaching a “Naked” Boryl Anion: Amide Metathesis as a Route to Calcium, Strontium, and Potassium Boryl Complexes
Source: Angew Chem Int Ed Engl. 2020 Nov 23;60(4):2064–8. doi: 10.1002/anie.202011839 (PMC7894291; doi:10.1002/anie.202011839)

checkCIF/PLATON report

You have not supplied any structure factors. As a result the full set of tests cannot be run.

THIS REPORT IS FOR GUIDANCE ONLY. IF USED AS PART OF A REVIEW PROCEDURE FOR PUBLICATION, IT SHOULD NOT REPLACE THE EXPERTISE OF AN EXPERIENCED CRYSTALLOGRAPHIC REFEREE.

No syntax errors found.   CIF dictionary   Interpreting this report

Datablock: 120apdv09

```
Bond precision:  C-C = 0.0049 A          Wavelength=0.71073

Cell:           a=21.8612(1)    b=21.5454(1)        c=22.0805(2)
                alpha=90        beta=118.8134(3)    gamma=90
Temperature:    150 K

                Calculated                  Reported
Volume          9112.51(10)                 9112.50(11)
Space group     P 21/c                      P 1 21/c 1
Hall group      -P 2ybc                     ?
Moiety formula  C40 H70 B Ca N3 O2 Si2      C40 H70 B1 Ca1 N3 O2 Si2
Sum formula     C40 H70 B Ca N3 O2 Si2      C40 H70 B1 Ca1 N3 O2 Si2
Mr              732.06                      732.08
Dx,g cm-3       1.067                       1.067
Z               8                           8
Mu (mm-1)       0.223                       0.223
F000            3200.0                      3200.0
F000’           3204.03
h,k,lmax        28,27,28                    28,27,28
Nref            20904                       20666
Tmin,Tmax       0.935,0.965                 0.950,0.960
Tmin’           0.913

Correction method= # Reported T Limits: Tmin=0.950 Tmax=0.960
AbsCorr = MULTI-SCAN

Data completeness= 0.989          Theta(max)= 27.487

R(reflections)= 0.0678( 13231)    wR2(reflections)= 0.1869( 20666)

S = 0.958                  Npar= 1003
```

The following ALERTS were generated. Each ALERT has the format
test-name_ALERT_alert-type_alert-level.
Click on the hyperlinks for more details of the test.

Alert level C

```
PLAT220_ALERT_2_C NonSolvent   Resd 1  C   Ueq(max)/Ueq(min) Range        3.8 Ratio
PLAT220_ALERT_2_C NonSolvent   Resd 2  C   Ueq(max)/Ueq(min) Range        3.1 Ratio
PLAT242_ALERT_2_C Low    'MainMol' Ueq as Compared to Neighbors of       Si62 Check
PLAT242_ALERT_2_C Low    'MainMol' Ueq as Compared to Neighbors of        C78 Check
PLAT242_ALERT_2_C Low    'MainMol' Ueq as Compared to Neighbors of        C32 Check
PLAT242_ALERT_2_C Low    'MainMol' Ueq as Compared to Neighbors of        C47 Check
```

Alert level G

```
PLAT002_ALERT_2_G Number of Distance or Angle Restraints on AtSite         40 Note
PLAT083_ALERT_2_G SHELXL Second Parameter in WGHT  Unusually Large      11.76 Why ?
PLAT142_ALERT_4_G s.u. on b - Axis Small or Missing .............     0.00010 Ang.
PLAT143_ALERT_4_G s.u. on c - Axis Small or Missing .............     0.00020 Ang.
PLAT145_ALERT_4_G s.u. on beta     Small or Missing .............      0.0003 Degree
PLAT175_ALERT_4_G The CIF-Embedded .res File Contains SAME Records          2 Report
PLAT177_ALERT_4_G The CIF-Embedded .res File Contains DELU Records          2 Report
PLAT178_ALERT_4_G The CIF-Embedded .res File Contains SIMU Records          2 Report
PLAT301_ALERT_3_G Main Residue  Disorder ..............(Resd  1  )        20% Note
PLAT301_ALERT_3_G Main Residue  Disorder ..............(Resd  2  )         6% Note
PLAT720_ALERT_4_G Number of Unusual/Non-Standard Labels ..........         50 Note
PLAT764_ALERT_4_G Overcomplete CIF Bond List Detected (Rep/Expd) .       1.10 Ratio
PLAT769_ALERT_4_G CIF Embedded explicitly supplied scattering data     Please Note
PLAT860_ALERT_3_G Number of Least-Squares Restraints .............        888 Note
PLAT882_ALERT_1_G No Datum for _diffrn_reflns_av_unetI/netI ......     Please Do !
```

```
   0 ALERT level A = Most likely a serious problem - resolve or explain
   0 ALERT level B = A potentially serious problem, consider carefully
   6 ALERT level C = Check. Ensure it is not caused by an omission or oversight
  15 ALERT level G = General information/check it is not something unexpected

   1 ALERT type 1 CIF construction/syntax error, inconsistent or missing data
   8 ALERT type 2 Indicator that the structure model may be wrong or deficient
   3 ALERT type 3 Indicator that the structure quality may be low
   9 ALERT type 4 Improvement, methodology, query or suggestion
   0 ALERT type 5 Informative message, check
```

It is advisable to attempt to resolve as many as possible of the alerts in all categories. Often the minor alerts point to easily fixed oversights, errors and omissions in your CIF or refinement strategy, so attention to these fine details can be worthwhile. In order to resolve some of the more serious problems it may be necessary to carry out additional measurements or structure refinements. However, the purpose of your study may justify the reported deviations and the more serious of these should normally be commented upon in the discussion or experimental section of a paper or in the "special_details" fields of the CIF. checkCIF was carefully designed to identify outliers and unusual parameters, but every test has its limitations and alerts that are not important in a particular case may appear. Conversely, the absence of alerts does not guarantee there are no aspects of the results needing attention. It is up to the individual to critically assess their own results and, if necessary, seek expert advice.

Publication of your CIF in IUCr journals

A basic structural check has been run on your CIF. These basic checks will be run on all CIFs submitted for publication in IUCr journals (*Acta Crystallographica*, *Journal of Applied Crystallography*, *Journal of Synchrotron Radiation*); however, if you intend to submit to *Acta Crystallographica Section C* or *E* or *IUCrData*, you should make sure that full publication checks are run on the final version of your CIF prior to submission.

Publication of your CIF in other journals

Please refer to the *Notes for Authors* of the relevant journal for any special instructions relating to CIF submission.

PLATON version of 10/08/2020; check.def file version of 06/08/2020

Datablock 120apdv09 - ellipsoid plot

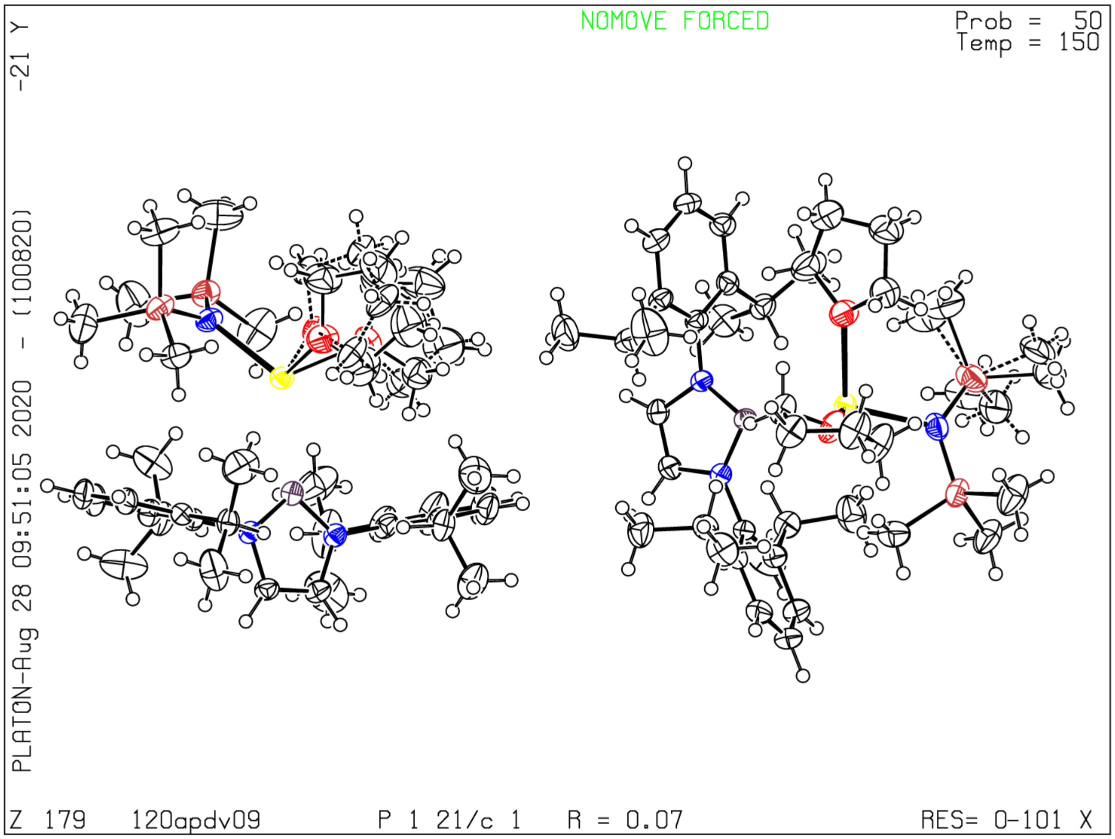

Supplement: Supplementary file 1 — Supplementary [file ANIE-60-2064-s001.zip › 1_checkcif.pdf]
