# Supplementary material for: Approaching a “Naked” Boryl Anion: Amide Metathesis as a Route to Calcium, Strontium, and Potassium Boryl Complexes
Source: Angew Chem Int Ed Engl. 2020 Nov 23;60(4):2064–8. doi: 10.1002/anie.202011839 (PMC7894291; doi:10.1002/anie.202011839)

checkCIF/PLATON report

You have not supplied any structure factors. As a result the full set of tests cannot be run.

THIS REPORT IS FOR GUIDANCE ONLY. IF USED AS PART OF A REVIEW PROCEDURE FOR PUBLICATION, IT SHOULD NOT REPLACE THE EXPERTISE OF AN EXPERIENCED CRYSTALLOGRAPHIC REFEREE.

No syntax errors found. CIF dictionary Interpreting this report

Datablock: 211apmaf18

```
Bond precision:  C-C = 0.0063 A          Wavelength=1.54184

Cell:            a=12.4841(2)     b=21.2203(3)       c=19.0889(3)
                 alpha=90         beta=94.791(1)     gamma=90
Temperature:     150 K

                Calculated                  Reported
Volume          5039.29(13)                 5039.29(13)
Space group     P 21/n                      P 21/n
Hall group      -P 2yn                      -P 2yn
Moiety formula  C44 H78 B N3 O3 Si2 Sr      C44 H78 B N3 O3 Si2 Sr
Sum formula     C44 H78 B N3 O3 Si2 Sr      C44 H78 B N3 O3 Si2 Sr
Mr              851.70                      851.70
Dx,g cm-3       1.123                       1.123
Z               4                           4
Mu (mm-1)       2.216                       2.216
F000            1832.0                      1832.0
F000'           1836.52
h,k,lmax        15,26,23                    15,26,23
Nref            10311                       10280
Tmin,Tmax       0.753,0.801                 0.807,1.000
Tmin'           0.683

Correction method= # Reported T Limits: Tmin=0.807 Tmax=1.000
AbsCorr = MULTI-SCAN

Data completeness= 0.997          Theta(max)= 74.491

R(reflections)= 0.0510( 8249)     wR2(reflections)= 0.1490( 10280)

S = 1.024                 Npar= 623
```

The following ALERTS were generated. Each ALERT has the format
test-name_ALERT_alert-type_alert-level.
Click on the hyperlinks for more details of the test.

Alert level C

```
PLAT220_ALERT_2_C NonSolvent   Resd 1  C   Ueq(max)/Ueq(min) Range        3.9 Ratio
PLAT230_ALERT_2_C Hirshfeld Test Diff for    C9       --C10      .        6.0 s.u.
PLAT241_ALERT_2_C High   'MainMol' Ueq as Compared to Neighbors of         C1 Check
PLAT241_ALERT_2_C High   'MainMol' Ueq as Compared to Neighbors of         C2 Check
PLAT241_ALERT_2_C High   'MainMol' Ueq as Compared to Neighbors of        C39 Check
PLAT242_ALERT_2_C Low    'MainMol' Ueq as Compared to Neighbors of        Sr1 Check
PLAT242_ALERT_2_C Low    'MainMol' Ueq as Compared to Neighbors of        Si2 Check
PLAT242_ALERT_2_C Low    'MainMol' Ueq as Compared to Neighbors of         C9 Check
PLAT242_ALERT_2_C Low    'MainMol' Ueq as Compared to Neighbors of        C12 Check
PLAT242_ALERT_2_C Low    'MainMol' Ueq as Compared to Neighbors of        C21 Check
PLAT242_ALERT_2_C Low    'MainMol' Ueq as Compared to Neighbors of        C24 Check
PLAT341_ALERT_3_C Low Bond Precision on  C-C Bonds ...............    0.00632 Ang.
PLAT360_ALERT_2_C Short  C(sp3)-C(sp3) Bond  C38      - C39      .       1.42 Ang.
```

Alert level G

```
PLAT002_ALERT_2_G Number of Distance or Angle Restraints on AtSite         29 Note
PLAT003_ALERT_2_G Number of Uiso or Uij Restrained non-H Atoms ...         27 Report
PLAT172_ALERT_4_G The CIF-Embedded .res File Contains DFIX Records          2 Report
PLAT176_ALERT_4_G The CIF-Embedded .res File Contains SADI Records          7 Report
PLAT178_ALERT_4_G The CIF-Embedded .res File Contains SIMU Records          3 Report
PLAT187_ALERT_4_G The CIF-Embedded .res File Contains RIGU Records          3 Report
PLAT230_ALERT_2_G Hirshfeld Test Diff for    Si1A     --N3       .        7.0 s.u.
PLAT301_ALERT_3_G Main Residue  Disorder ..............(Resd  1  )        24% Note
PLAT412_ALERT_2_G Short Intra XH3 .. XHn     H10B     ..H33A     .       1.97 Ang.
                                                      x,y,z  =      1_555 Check
PLAT412_ALERT_2_G Short Intra XH3 .. XHn     H13B     ..H27F     .       1.96 Ang.
                                                      x,y,z  =      1_555 Check
PLAT412_ALERT_2_G Short Intra XH3 .. XHn     H22B     ..H44D     .       2.03 Ang.
                                                      x,y,z  =      1_555 Check
PLAT412_ALERT_2_G Short Intra XH3 .. XHn     H31C     ..H27E     .       2.09 Ang.
                                                      x,y,z  =      1_555 Check
PLAT860_ALERT_3_G Number of Least-Squares Restraints .............        553 Note
PLAT941_ALERT_3_G Average HKL Measurement Multiplicity ...........        2.4 Low
PLAT965_ALERT_2_G The SHELXL WEIGHT Optimisation has not Converged     Please Check
```

---

0 **ALERT level A** = Most likely a serious problem - resolve or explain
0 **ALERT level B** = A potentially serious problem, consider carefully
13 **ALERT level C** = Check. Ensure it is not caused by an omission or oversight
15 **ALERT level G** = General information/check it is not something unexpected

0 ALERT type 1 CIF construction/syntax error, inconsistent or missing data
20 ALERT type 2 Indicator that the structure model may be wrong or deficient
4 ALERT type 3 Indicator that the structure quality may be low
4 ALERT type 4 Improvement, methodology, query or suggestion
0 ALERT type 5 Informative message, check

It is advisable to attempt to resolve as many as possible of the alerts in all categories. Often the minor alerts point to easily fixed oversights, errors and omissions in your CIF or refinement strategy, so attention to these fine details can be worthwhile. In order to resolve some of the more serious problems it may be necessary to carry out additional measurements or structure refinements. However, the purpose of your study may justify the reported deviations and the more serious of these should normally be commented upon in the discussion or experimental section of a paper or in the "special_details" fields of the CIF. checkCIF was carefully designed to identify outliers and unusual parameters, but every test has its limitations and alerts that are not important in a particular case may appear. Conversely, the absence of alerts does not guarantee there are no aspects of the results needing attention. It is up to the individual to critically assess their own results and, if necessary, seek expert advice.

Publication of your CIF in IUCr journals

A basic structural check has been run on your CIF. These basic checks will be run on all CIFs submitted for publication in IUCr journals (*Acta Crystallographica*, *Journal of Applied Crystallography*, *Journal of Synchrotron Radiation*); however, if you intend to submit to *Acta Crystallographica Section C* or *E* or *IUCrData*, you should make sure that full publication checks are run on the final version of your CIF prior to submission.

Publication of your CIF in other journals

Please refer to the *Notes for Authors* of the relevant journal for any special instructions relating to CIF submission.

PLATON version of 10/08/2020; check.def file version of 06/08/2020

Datablock 211apmaf18 - ellipsoid plot

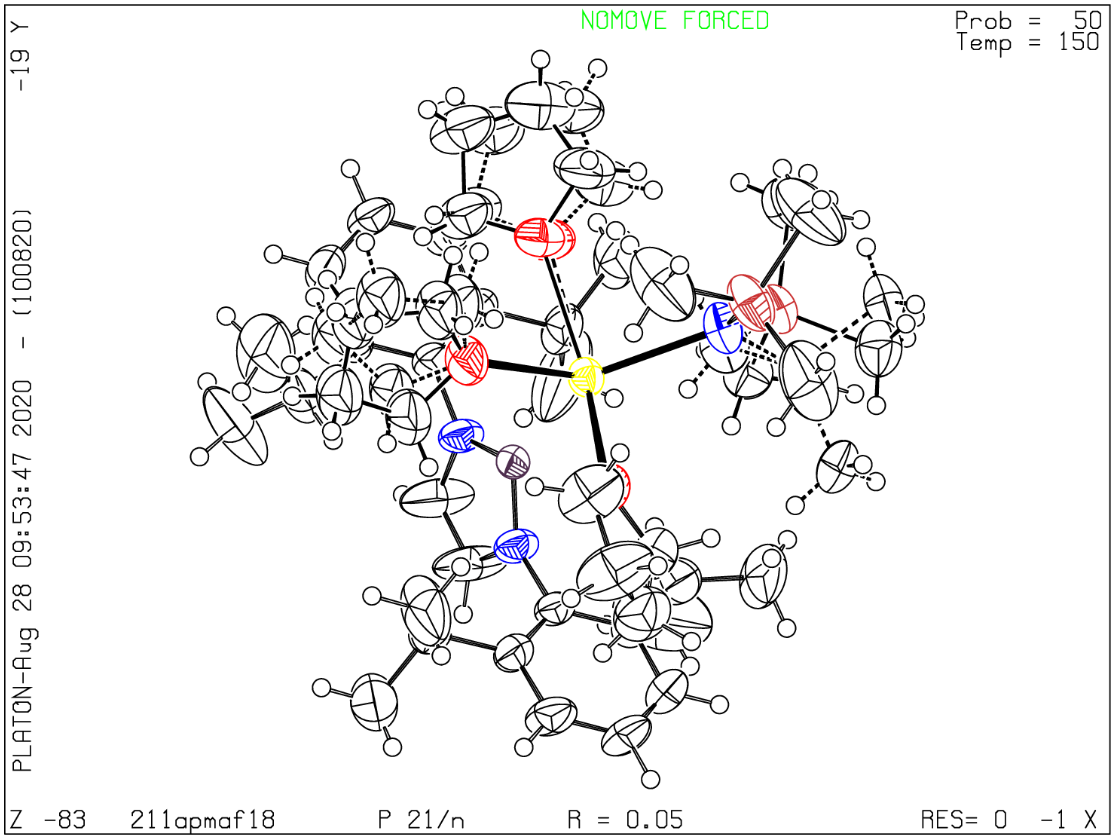

Supplement: Supplementary file 1 — Supplementary [file ANIE-60-2064-s001.zip › 2_checkcif.pdf]
