# Supplementary material for: Approaching a “Naked” Boryl Anion: Amide Metathesis as a Route to Calcium, Strontium, and Potassium Boryl Complexes
Source: Angew Chem Int Ed Engl. 2020 Nov 23;60(4):2064–8. doi: 10.1002/anie.202011839 (PMC7894291; doi:10.1002/anie.202011839)

checkCIF/PLATON report

You have not supplied any structure factors. As a result the full set of tests cannot be run.

THIS REPORT IS FOR GUIDANCE ONLY. IF USED AS PART OF A REVIEW PROCEDURE FOR PUBLICATION, IT SHOULD NOT REPLACE THE EXPERTISE OF AN EXPERIENCED CRYSTALLOGRAPHIC REFEREE.

No syntax errors found. CIF dictionary Interpreting this report

Datablock: 232apmaf19

```
Bond precision:  C-C = 0.0031 A          Wavelength=1.54184

Cell:           a=14.7846(3)    b=21.4971(5)       c=19.8902(4)
                alpha=90        beta=100.010(2)    gamma=90
Temperature:    150 K
```

	Calculated	Reported
Volume	6225.4(2)	6225.4(2)
Space group	I 2/a	I 2/a
Hall group	-I 2ya	-I 2ya
Moiety formula	C52 H72 B2 K2 N4, 2(C7 H14)	C52 H72 B2 K2 N4, 2(C7 H14)
Sum formula	C66 H100 B2 K2 N4	C66 H100 B2 K2 N4
Mr	1049.32	1049.31
Dx,g cm-3	1.120	1.120
Z	4	4
Mu (mm-1)	1.643	1.643
F000	2288.0	2288.0
F000’	2296.13	
h,k,lmax	18,26,24	18,26,24
Nref	6386	6333
Tmin,Tmax	0.848,0.877	0.727,1.000
Tmin’	0.848	

```
Correction method= # Reported T Limits: Tmin=0.727 Tmax=1.000
AbsCorr = MULTI-SCAN

Data completeness= 0.992          Theta(max)= 74.501

R(reflections)= 0.0459( 4952)     wR2(reflections)= 0.1322( 6333)

S = 1.041                  Npar= 347
```

The following ALERTS were generated. Each ALERT has the format
test-name_ALERT_alert-type_alert-level.
Click on the hyperlinks for more details of the test.

Alert level C

```
PLAT410_ALERT_2_C Short Intra H...H Contact  H1A      ..H21      .       1.96 Ang.
                                                      x,y,z  =      1_555 Check
```

Alert level G

```
PLAT231_ALERT_4_G Hirshfeld Test (Solvent)   C28      --C33      .        5.5 s.u.
PLAT343_ALERT_2_G Unusual          Angle Range in Main Residue for         C1 Check
PLAT343_ALERT_2_G Unusual          Angle Range in Main Residue for         C2 Check
PLAT764_ALERT_4_G Overcomplete CIF Bond List Detected (Rep/Expd) .       1.16 Ratio
PLAT779_ALERT_4_G Suspect or Irrelevant (Bond) Angle(s) in CIF . #        197 Check
              K1   -C21  -H21     2.556   1.555   1.555             35.80 Deg.
PLAT941_ALERT_3_G Average HKL Measurement Multiplicity ...........        2.9 Low
```

---

0 **ALERT level A** = Most likely a serious problem - resolve or explain
0 **ALERT level B** = A potentially serious problem, consider carefully
1 **ALERT level C** = Check. Ensure it is not caused by an omission or oversight
6 **ALERT level G** = General information/check it is not something unexpected

0 ALERT type 1 CIF construction/syntax error, inconsistent or missing data
3 ALERT type 2 Indicator that the structure model may be wrong or deficient
1 ALERT type 3 Indicator that the structure quality may be low
3 ALERT type 4 Improvement, methodology, query or suggestion
0 ALERT type 5 Informative message, check

It is advisable to attempt to resolve as many as possible of the alerts in all categories. Often the minor alerts point to easily fixed oversights, errors and omissions in your CIF or refinement strategy, so attention to these fine details can be worthwhile. In order to resolve some of the more serious problems it may be necessary to carry out additional measurements or structure refinements. However, the purpose of your study may justify the reported deviations and the more serious of these should normally be commented upon in the discussion or experimental section of a paper or in the "special_details" fields of the CIF. checkCIF was carefully designed to identify outliers and unusual parameters, but every test has its limitations and alerts that are not important in a particular case may appear. Conversely, the absence of alerts does not guarantee there are no aspects of the results needing attention. It is up to the individual to critically assess their own results and, if necessary, seek expert advice.

Publication of your CIF in IUCr journals

A basic structural check has been run on your CIF. These basic checks will be run on all CIFs submitted for publication in IUCr journals (*Acta Crystallographica*, *Journal of Applied Crystallography*, *Journal of Synchrotron Radiation*); however, if you intend to submit to *Acta Crystallographica Section C* or *E* or *IUCrData*, you should make sure that full publication checks are run on the final version of your CIF prior to submission.

Publication of your CIF in other journals

Please refer to the *Notes for Authors* of the relevant journal for any special instructions relating to CIF submission.

PLATON version of 10/08/2020; check.def file version of 06/08/2020

Datablock 232apmaf19 - ellipsoid plot

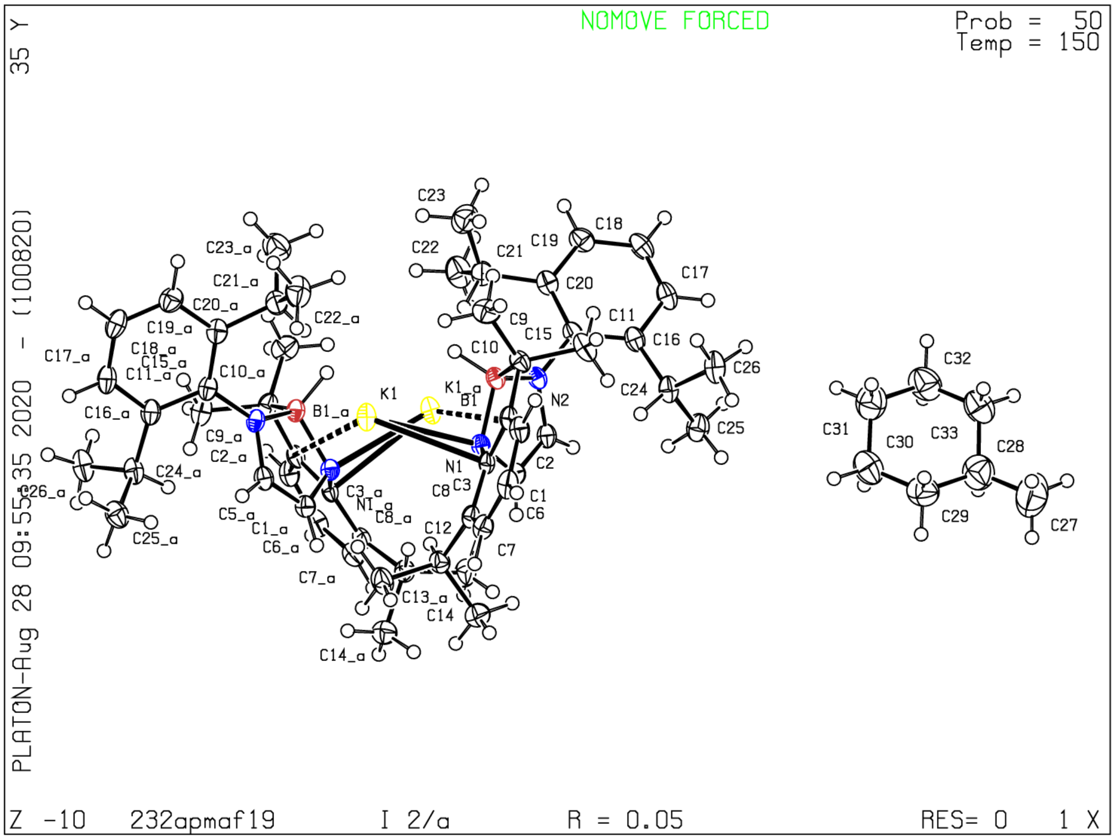

Supplement: Supplementary file 1 — Supplementary [file ANIE-60-2064-s001.zip › 4_checkcif.pdf]
