# Supplementary material for: Approaching a “Naked” Boryl Anion: Amide Metathesis as a Route to Calcium, Strontium, and Potassium Boryl Complexes
Source: Angew Chem Int Ed Engl. 2020 Nov 23;60(4):2064–8. doi: 10.1002/anie.202011839 (PMC7894291; doi:10.1002/anie.202011839)
Supplement: Supplementary file 2 — Supplementary [file ANIE-60-2064-s002.pdf]

Angewandte Chemie
Eine Zeitschrift der Gesellschaft Deutscher Chemiker

Supporting Information

Approaching a "Naked" Boryl Anion: Amide Metathesis as a Route to Calcium, Strontium, and Potassium Boryl Complexes

*Andrey V. Protchenko, Petra Vasko, M. Ángeles Fuentes, Jamie Hicks, Dragoslav Vidovic, and Simon Aldridge**

anie_202011839_sm_miscellaneous_information.pdf
anie_202011839_sm_cif.zip

Supporting Information

Table of Contents

1. General methods and instrumentation s3
2. Syntheses of novel compounds s4
3. Crystallographic data s13
4. Computational details s14
5. References s18

1. General methods and instrumentation

All manipulations were carried out using standard Schlenk line or dry-box techniques under an atmosphere of argon or dinitrogen. Solvents were degassed by sparging with dinitrogen and dried by passing through a column of the appropriate drying agent. THF and Et_2O were refluxed over sodium-potassium alloy, distilled and stored over sodium mirror. NMR spectra were measured in C_6D_6 which was dried over potassium, distilled under reduced pressure and stored under dinitrogen in Teflon valve ampoules. NMR samples were prepared under dinitrogen in 5 mm Wilmad 507-PP tubes fitted with J. Young Teflon valves. ^{1}H, $^{13}C\{^{1}H\}$ and $^{11}B\{^{1}H\}$ NMR spectra were recorded on a Bruker Avance III HD nanobay (400 MHz) and referenced internally to residual protio-solvent (^{1}H) or solvent (^{13}C) resonances and are reported relative to tetramethylsilane (δ = 0 ppm), while ^{11}B NMR spectra were referenced to external $BF_3{\cdot}OEt_2$. Assignments were confirmed using two dimensional ^{1}H-^{1}H and ^{13}C-^{1}H NMR correlation experiments. Chemical shifts are quoted in δ (ppm) and coupling constants in Hz. Starting materials $M\{N(SiMe_3)_2\}_2(thf)_2$ (M = Ca, Sr, Ba) were prepared from MBr_2 (M = Ca, Sr) or $Ba(OTf)_2$ by salt metathesis with $K\{N(SiMe_3)_2\}$ in thf and recrystallised from hexane;[s1] $Li\{B(NDippCH)_2\}(thf)_2$ (**I**)[s2] was prepared by the literature procedure.[s2]

2. Syntheses of novel compounds

Reaction of Mg{N(SiMe$_3$)$_2$}$_2$ with I. *NMR scale test reaction*: Unsolvated Mg{N(SiMe$_3$)$_2$}$_2$ (0.022 g, 0.064 mmol) was dissolved in C_6D_6 (0.5 mL) showing two ^{1}H NMR signals (0.44 and 0.36 ppm). To this solution solid Li{B(NDippCH)$_2$}(thf)$_2$ (**I**) (0.026 g, 0.048 mmol) was added forming single new boryl species with CH backbone signal at 6.44 ppm (δ(^{11}B) 35.1 ppm), which showed no sign of decomposition upon storage at room temperature for several days. *Attempted synthesis of [Mg{B(NDippCH)$_2$}{N(SiMe$_3$)$_2$}(thf)]*: Benzene (10 mL) was added to a mixture of Li{B(NDippCH)$_2$}(thf)$_2$ (**I**) (0.202 g, 0.37 mmol) and Mg{N(SiMe$_3$)$_2$}$_2$ (0.128 g, 0.37 mmol) at room temperature; the mixture was stirred until the reagents had dissolved producing light yellow solution. The solvent was removed *in vacuo* leaving semi-crystalline material, which was dissolved in minimal amount of pentane and the solution was stored overnight at –30 °C yielding a lump of colourless crystalline product characterised by X-ray diffraction as co-crystal [Mg{B(NDippCH)$_2$}{N(SiMe$_3$)$_2$}(thf)]·[Li{N(SiMe$_3$)$_2$}(thf)]$_2$. Repeated crystallisation did not produce Li-free product.

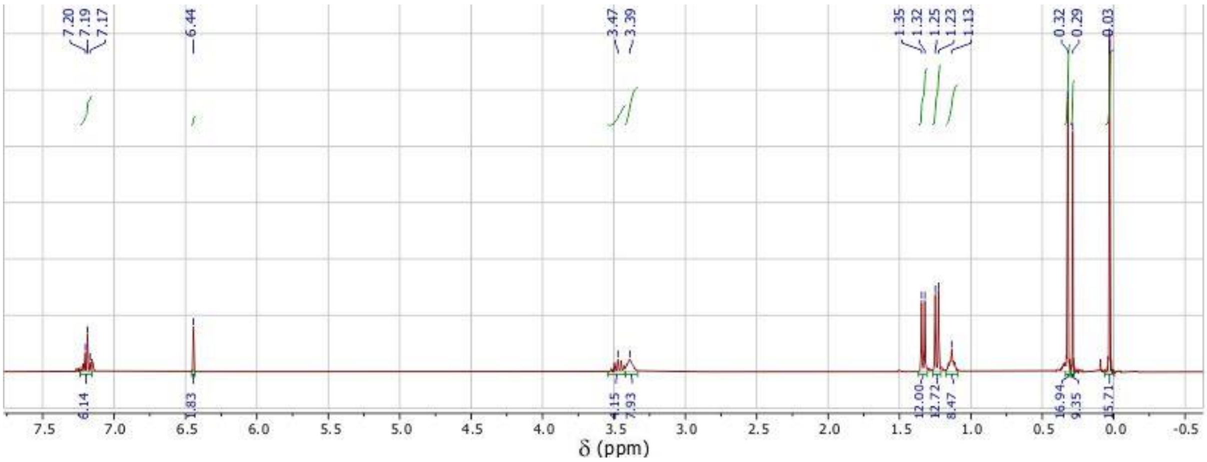

Figure s1: ^{1}H NMR spectrum of Mg{N(SiMe$_3$)$_2$}$_2$ + **I** in C_6D_6

[Ca{B(NDippCH)$_2$}{N(SiMe$_3$)$_2$}(thf)$_2$] (1): *NMR scale reaction*: Solid Li{B(NDippCH)$_2$}(thf)$_2$ (**I**) (0.017 g, 0.032 mmol) was added to a solution of Ca{N(SiMe$_3$)$_2$}$_2$(thf)$_2$ (0.018 g, 0.035 mmol) in C_6D_6 (0.5 mL). ^{1}H NMR spectrum showed formation of single new boryl species with CH backbone signal at 6.44 ppm (δ(^{11}B) 41.6 ppm), which decomposed by more than 50% overnight at room temperature. *Bulk synthesis of **1***: Diethyl ether (10 mL) was added to a mixture of Li{B(NDippCH)$_2$}(thf)$_2$ (**I**) (0.220 g, 0.41 mmol) and Ca{N(SiMe$_3$)$_2$}$_2$(thf)$_2$ (0.205 g, 0.41 mmol) at –30 °C; the mixture was stirred until the reagents had dissolved completely (ca. 30 min). The solvent was removed *in vacuo* leaving yellow semi-crystalline honey-like material. The residue was dissolved in pentane (ca. 10 mL) and the solution was concentrated *in vacuo* until crystallisation started. Storing the solution overnight at –30 °C yielded large pale yellow blocks of **1** (0.130 g, 0.18 mmol, 43%). **1** decomposes in benzene solution at room temperature over a period of 12-24 h to give HB(NDippCH)$_2$ and Ph B(NDippCH)$_2$. ^{1}H NMR (C_6D_6): δ 7.22 (m, 6H, *m*- and *p*-H of Ar), 6.47 (s, 2H, NCH), 3.69 (sept, ^3J = 6.9 Hz, 4H, C*H*Me$_2$), 3.22 (m, 8H, α-CH$_2$ of thf), 1.38 (d, ^3J = 6.9 Hz, 12H, CH*Me*$_2$), 1.30 (d, ^3J = 6.9 Hz, 12H, CH*Me*$_2$), 1.19 (m, 8H, β-CH$_2$ of thf), 0.12 (s, 18H, SiMe$_3$). ^{13}C NMR (C_6D_6): δ 147.0 (*o*-C of Ar), 146.2 (*ipso*-C of Ar), 126.0 (*p*-CH of Ar), 123.1 (*m*-CH of Ar), 120.0 (NCH), 68.2 (α-CH$_2$ of thf), 28.5 (*C*HMe$_2$), 25.6 (CH*Me*$_2$), 25.0 (β-CH$_2$ of thf), 24.5 (CH*Me*$_2$), 5.8 (s, SiMe$_3$). ^{11}B NMR (C_6D_6): δ 41.2 (br).

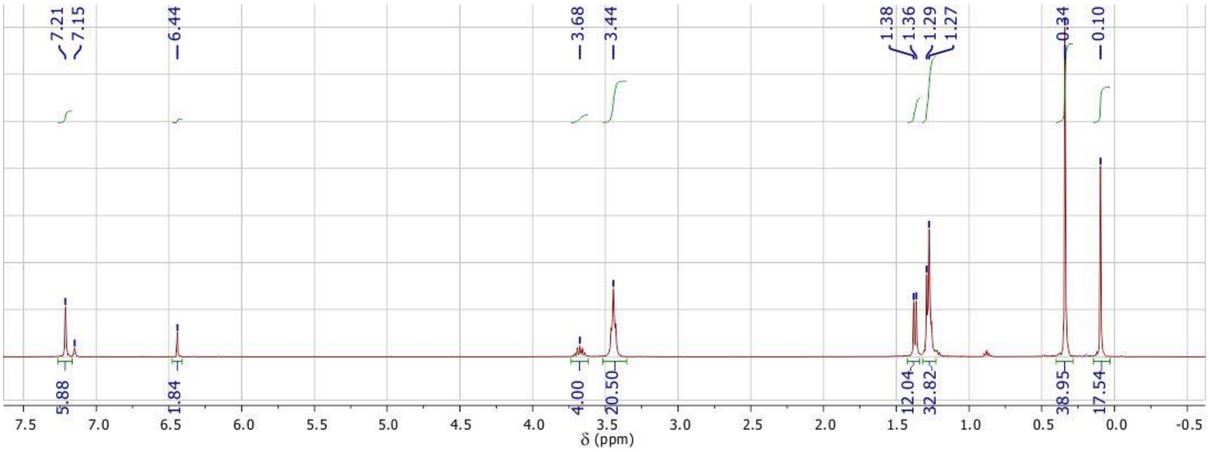

Figure s2: ^{1}H NMR spectrum of Ca{N(SiMe$_3$)$_2$}$_2$(thf)$_2$ + **I** in C_6D_6

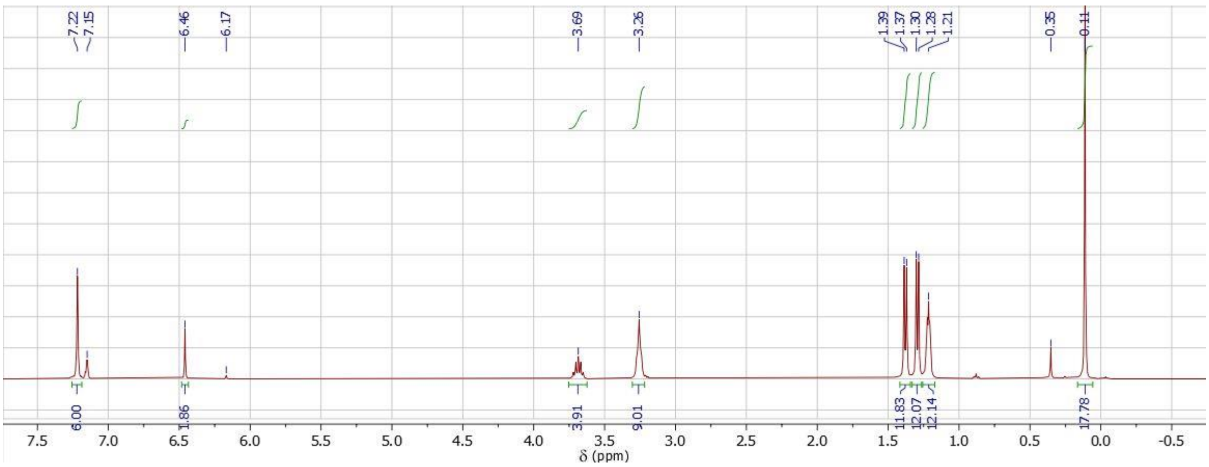

Figure s3: ^{1}H NMR spectrum of **1** in C_6D_6

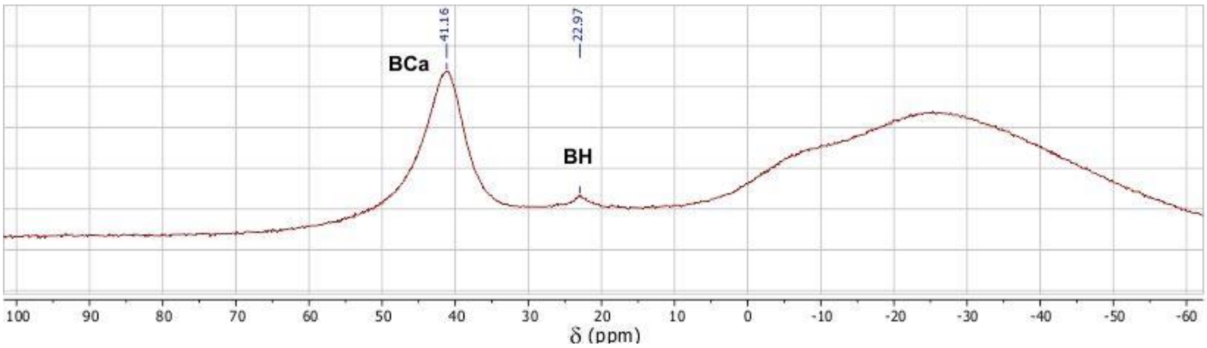

Figure s4: ^{11}B NMR spectrum of **1** in C_6D_6

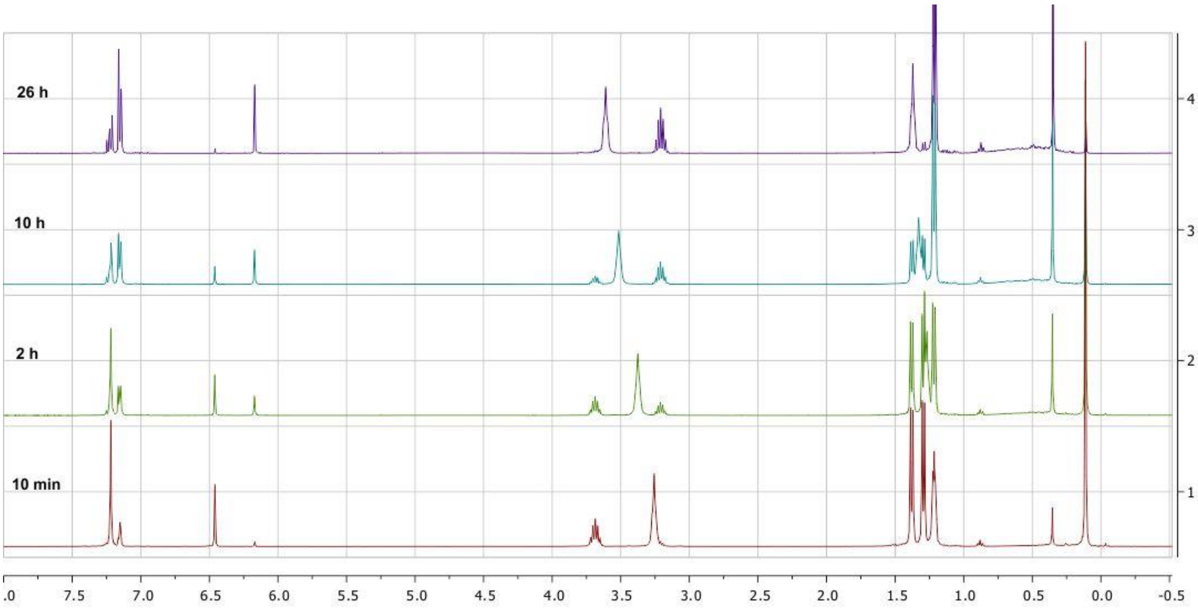

Figure s5: Decomposition of **1** in C_6D_6 followed by ^{1}H NMR spectra at 298 K

Sr{B(NDippCH)$_2$}{N(SiMe$_3$)$_2$}(thf)$_3$ (2): *NMR scale reaction*: Solid Li{B(NDippCH)$_2$}(thf)$_2$ (**I**) (0.021 g, 0.039 mmol) was added to a solution of Sr{N(SiMe$_3$)$_2$}$_2$(thf)$_2$ (0.022 g, 0.040 mmol) in C_6D_6 (0.5 mL). ^{1}H NMR spectrum showed formation of one major new boryl species with CH backbone signal at 6.48 ppm (δ(^{11}B) 45.7 ppm), which decomposed completely at room temperature overnight. *Bulk synthesis of* ***2***: Diethyl ether (0.5 mL) was vacuum-transferred to a mixture of Li{B(NDippCH)$_2$}(thf)$_2$ (**I**) (21 mg, 0.040 mmol) and Sr{N(SiMe$_3$)$_2$}$_2$(thf)$_2$ (22 mg, 0.040 mmol) in a two-section tube at –196 °C (liquid N_2); the mixture was briefly warmed up to room temperature and shaken by hand until the reagents had dissolved completely. All volatiles were removed *in vacuo*, pentane (0.5 mL) was added by vacuum transfer and the tube was sealed. The orange-yellow solution was concentrated to a drop of oil and the tube was stored at –30 °C for three days yielding pale yellow blocks of **2** suitable for X-ray diffraction (15 mg, ca. 40%). **2** decomposes in benzene solution at room temperature over a period of 3-6 h to give HB(NDippCH)$_2$ and PhB(NDippCH)$_2$. ^{1}H NMR (C_6D_6): δ 7.22 (m, 6H, *m*- and *p*-H of Ar), 6.49 (s, 2H, NCH), 3.73 (sept, ^3J = 6.9 Hz, 4H, C*H*Me$_2$), 3.51 (m, 8H, α-CH_2 of thf), 1.39 (d, ^3J = 6.9 Hz, 12H, CH*Me*$_2$), 1.31 (d, ^3J = 6.9 Hz, 12H, CH*Me*$_2$), 1.30 (m, 8H, β-CH_2 of thf), 0.14 (s, 18H, SiMe$_3$). ^{11}B NMR (C_6D_6): δ 45.1 (br).

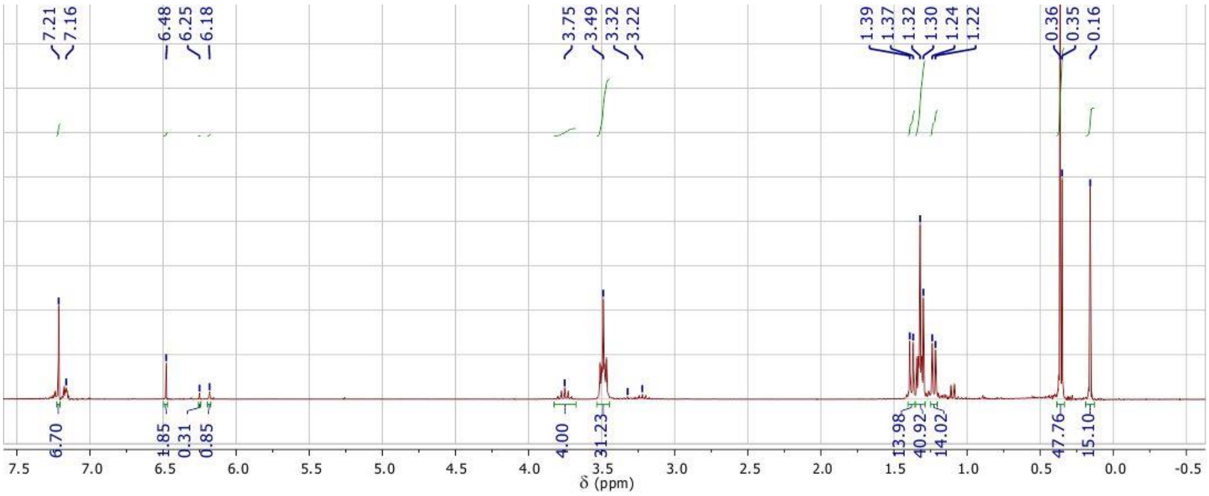

Figure s6: ^{1}H NMR spectrum of Sr{N(SiMe$_3$)$_2$}$_2$(thf)$_2$ + **I** in C_6D_6

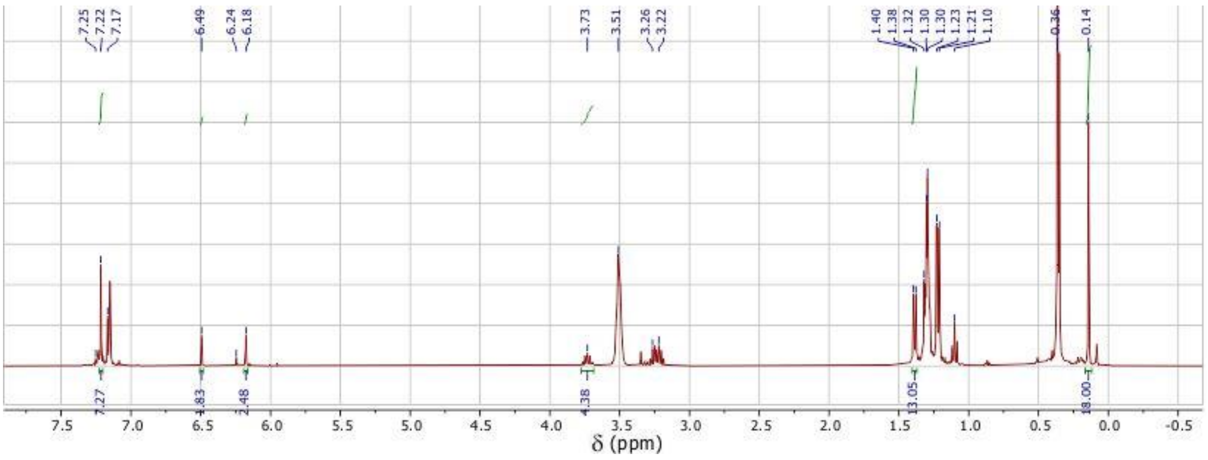

Figure s7: ^{1}H NMR spectrum: decomposition of **2** in C_6D_6

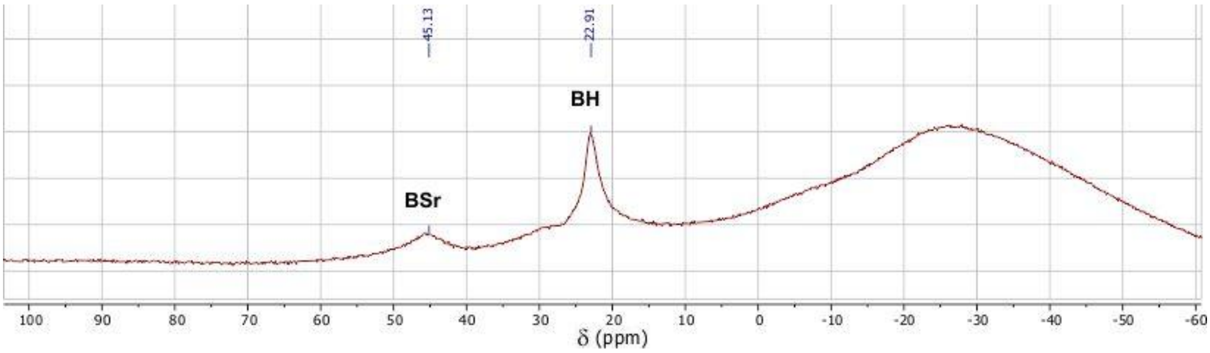

Figure s8: ^{11}B NMR spectrum: decomposition of **2** in C_6D_6

Reaction of Ba{N(SiMe$_3$)$_2$}$_2$(thf)$_2$ with I. Solid Li{B(NDippCH)$_2$}(thf)$_2$ (**I**) (0.032 g, 0.059 mmol) was added to the solution of Ba{N(SiMe$_3$)$_2$}$_2$(thf)$_2$ (0.036 g, 0.059 mmol) in C_6D_6 (0.5 mL). ^{1}H NMR spectrum showed only formation of decomposition products with CH backbone signals at 6.25 (PhB(NDippCH)$_2$) and 6.18 ppm (HB(NDippCH)$_2$).

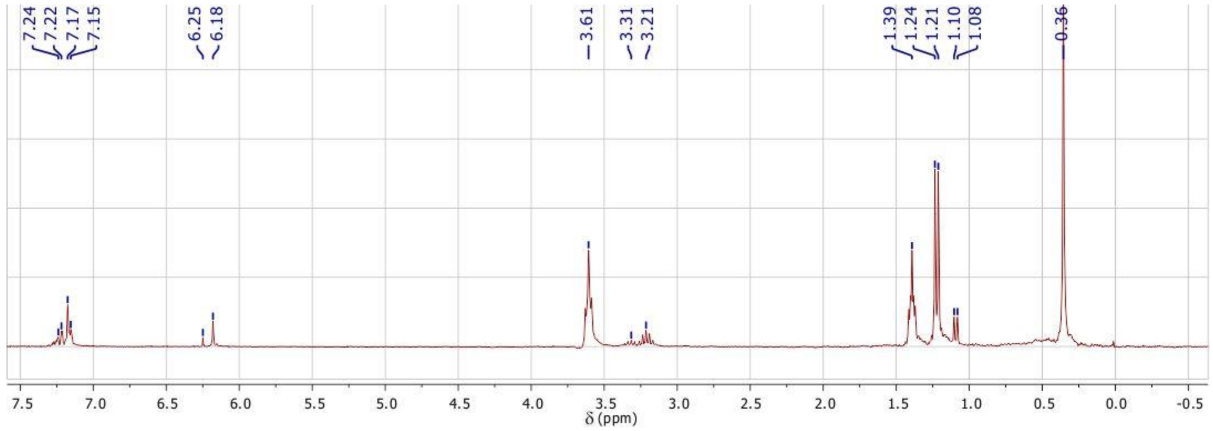

Figure s4: ^{1}H NMR spectrum of Ba{N(SiMe$_3$)$_2$}$_2$(thf)$_2$ + **I** in C_6D_6

[K{B(NDippCH)$_2$}]$_2$ (3): Hexane (0.5 mL) was vacuum-transferred to a mixture of Li{B(NDippCH)$_2$}(thf)$_2$ (**I**) (33 mg, 0.061 mmol) and K{N(SiMe$_3$)$_2$} (13 mg, 0.065 mmol) in a two-section tube at –196 °C (liquid N$_2$) and the tube was sealed; the mixture was briefly warmed up to room temperature and sonicated until the reagents had dissolved completely. The tube was immediately cooled to –30 °C, the orange-yellow solution was concentrated to ½ volume (by applying dry ice to the empty part) and the tube was stored at –30 °C for two days yielding deep yellow blocks (larger crystals look orange) of [K{B(NDippCH)$_2$}]$_2$ suitable for X-ray diffraction (13 mg, MW = 426.50 (monomer), 0.030 mmol, 50%). Dissolving in C$_6$D$_6$ gave a deep yellow solution, which started darkening immediately and turned red after ^{1}H and ^{11}B NMR spectra have been measured (~10 min). **3** also decomposes in the solid state over a period of 48 h at room temperature. Accordingly, clean NMR and microanalytical data on **3** could not be obtained. Its composition is supported, however, by the isolation and characterization of **4** (vide infra), the product of its decomposition by intramolecular C-H insertion.

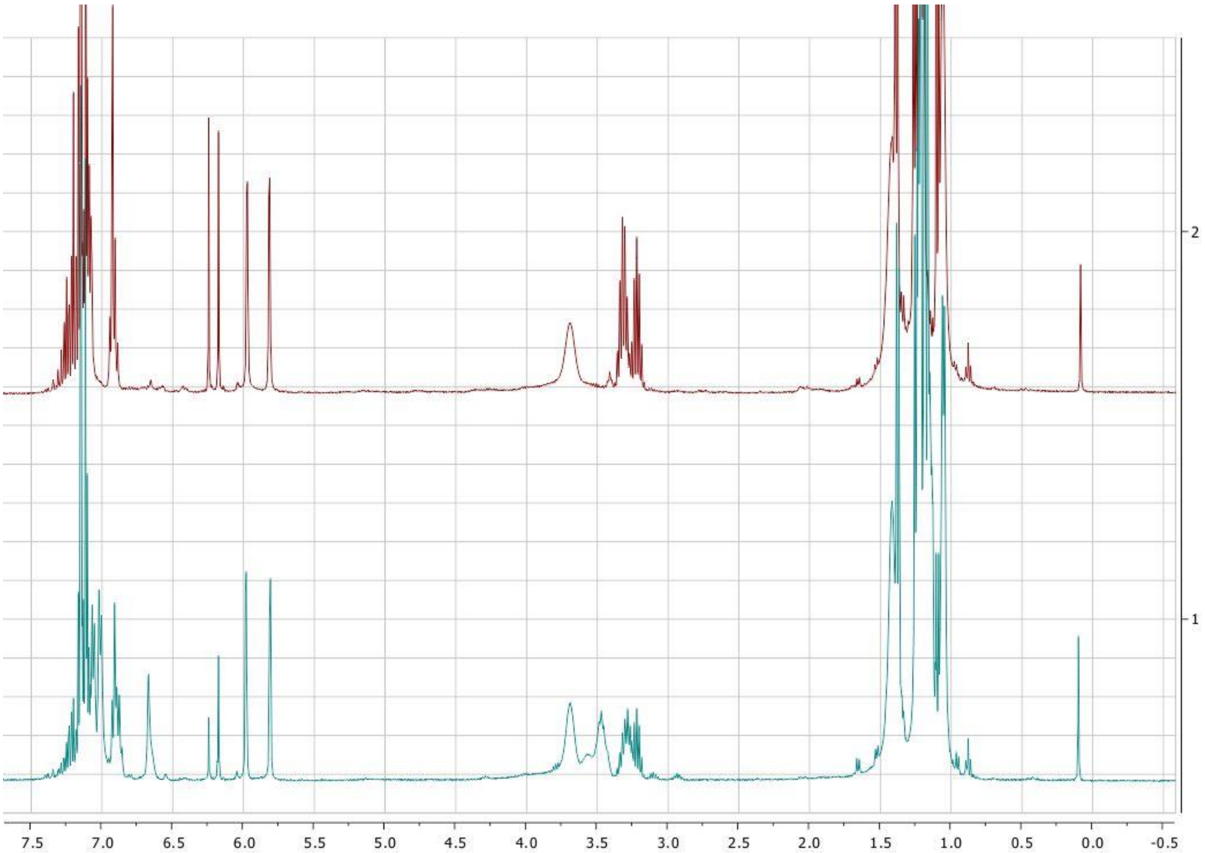

Figure s10: Decomposition of **3** in C$_6$D$_6$ followed by ^{1}H NMR at 298 K: after 5 min (1); after 1 h (2) (peak at 6.69 ppm is assigned to remaining **3** while peaks at 6.18 and 6.25 ppm correspond to HB(NDippCH)$_2$ and PhB(NDippCH)$_2$, respectively)

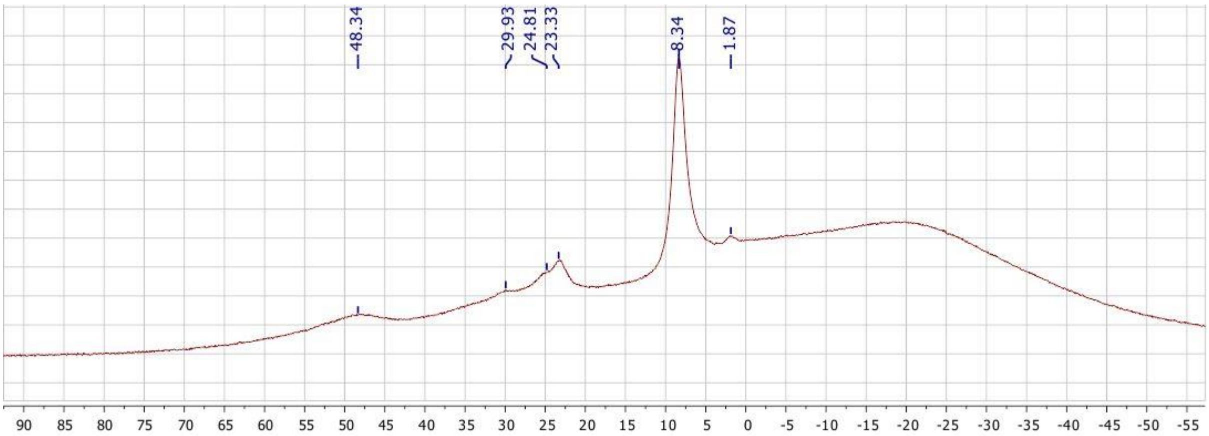

Figure s11: ^{11}B NMR spectrum: decomposition of **3** in C_6D_6 (broad peak at 48.3 ppm is assigned to remaining **3**)

$[K\{HB(CMe_2C_6H_3(CHMe_2)NCHCHNDipp)\}]_n$ (4): The above sample of decomposed **3** was transferred to a crystallisation tube and evaporated to dryness. Addition of hexane (0.3 mL) resulted in formation of white microcrystalline solid, which was washed with a small amount of cold hexane and dried *in vacuo*. NMR spectroscopy showed that the solid was almost pure **4** (containing ~0.5 molecule of hexane per monomeric unit). Crystals suitable for X-ray diffraction were obtained from methylcyclohexane solution. Yield ca. 10 mg, 63% ^{1}H NMR (C_6D_6): δ 7.07-7.13 (m, 4H, CH of Ar), 6.89-6.94 (m, 2H, CH of Ar), 5.98 (d, ^{3}J = 2.1 Hz, 1H, NCH), 5.82 (d, ^{3}J = 2.1 Hz, 1H, NCH), 3.70 (br s, 2H, C*H*Me₂), 3.31 (sept, ^{3}J = 6.8 Hz, 1H, C*H*Me₂), 1.43 (br s, 6H, CH*Me*₂), 1.39 (d, ^{3}J = 6.8 Hz, 3H, CH*Me*₂), 1.26 (d, ^{3}J = 6.8 Hz, 3H, CH*Me*₂), 1.23 (s, 3H, C*Me*₂ + CH_2 of hexane), 1.20 (s, 3H, C*Me*₂), 1.06 (br s, 6H, CH*Me*₂), 0.88 (t, 3H, CH_3 of hexane). ^{11}B NMR (C_6D_6): δ 8.4 (br).

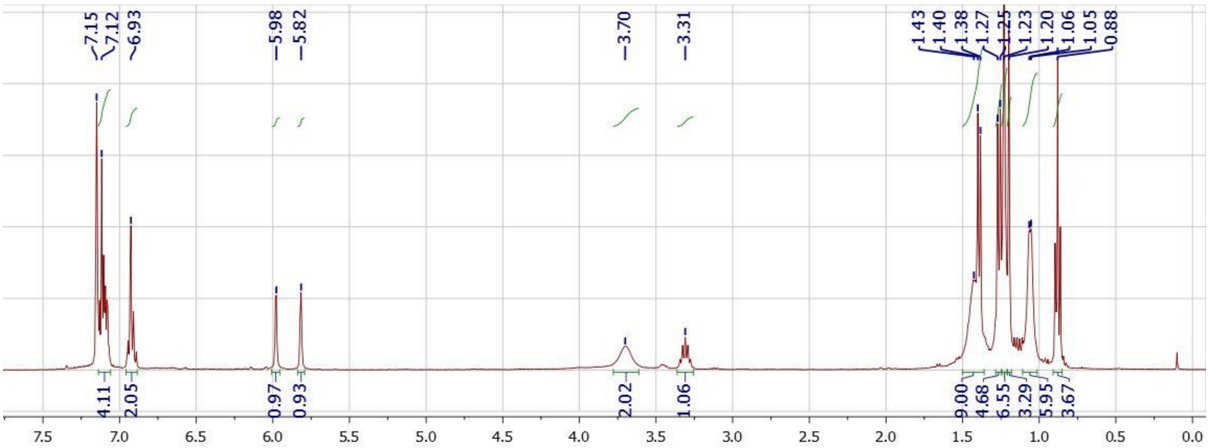

Figure s12: ^{1}H NMR spectrum of **4** in C_6D_6

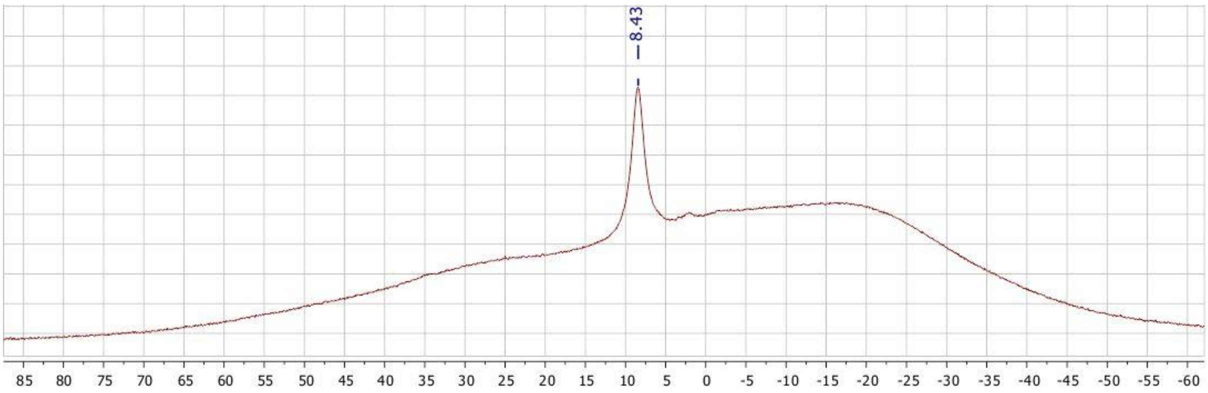

Figure s13: ^{11}B NMR spectrum of **4** in C_6D_6

3. Crystallographic data

1: $C_{40}H_{70}BCaN_3O_2Si_2$, monoclinic, *P*$2_1$/*c*, M_r = 732.08, *a* = 21.8612(1), *b* = 21.5454(1), *c* = 22.0805(2) Å, □ = 118.8134(3)°, V = 9112.50(11) Å^3, □$_x$ = 1.067 Mg m^{-3}, *Z* = 8, R_1 = 0.0678 (13231 observed reflections), wR_2 = 0.1869 (20666 total reflections). CCDC reference: 2021075.

2: $C_{44}H_{78}BN_3O_3Si_2Sr$, monoclinic, *P*$2_1$/*n*, M_r = 851.70, *a* = 12.4841(2), *b* = 21.2203(3), *c* = 19.0889(3) Å, □ = 94.7910(10)°, V = 5039.29(13) Å^3, □$_x$ = 1.123 Mg m^{-3}, *Z* = 4, R_1 = 0.0510 (8249 observed reflections), wR_2 = 0.1490 (10280 total reflections). CCDC reference: 2021078.

3: $C_{52}H_{72}B_2K_2N_4$, monoclinic, *P*2_1/*n*, M_r = 852.95, *a* = 10.6897(2), *b* = 18.7258(4), *c* = 12.9727(3) Å, □ = 94.133(2)°, V = 2590.03(10) Å^3, □$_x$ = 1.094 Mg m^{-3}, *Z* = 2, R_1 = 0.0442 (4699 observed reflections), wR_2 = 0.1259 (5364 total reflections). CCDC reference: 2021076.

4($2C_6H_{11}CH_3$): $C_{66}H_{100}B_2K_2N_4$, monoclinic, *I*2/*a*, M_r = 1049.31, *a* = 14.7846(3), *b* = 21.4971(5), *c* = 19.8902(4) Å, □ = 100.010(2)°, V = 6225.4(2) Å^3, □$_x$ = 1.120 Mg m^{-3}, *Z* = 4, R_1 = 0.0459 (4952 observed reflections), wR_2 = 0.1322 (6333 total reflections). CCDC reference: 2021077.

4. Computational details

The geometry optimizations were performed with the Gaussian16 (Revision C.01) programme [s3] using the PBE1PBE hybrid exchange functional [s4] and Def-TZVP basis set.[s5] In addition, Grimme's empirical dispersion correction with Becke-Johnson damping (GD3BJ) was used as well as an ultrafine integration grid.[s6] Full analytical frequency calculations were performed for the optimized structures to ensure the nature of the stationary points found (minima, no imaginary frequencies). Simplified model compounds **3'** and **I'** where the isopropyl groups were replaced by methyl groups were used for the QTAIM analysis (performed by the programme AIMAll, version 19.10.12) to reduce computational cost.[s7]

Xyz-coordinates of optimized compounds

3
132

K	-0.281379	-1.868483	-0.076631
N	-3.658645	-0.493441	-0.314208
N	-3.287865	1.609799	0.324987
C	-3.519784	-1.857461	-0.651594
C	-3.605102	-2.831368	0.359107
C	-3.296552	-4.151140	0.038508
H	-3.339596	-4.913082	0.808869
C	-2.928365	-4.505788	-1.251100
H	-2.682085	-5.536739	-1.481207
C	-2.890537	-3.543234	-2.247243
H	-2.620675	-3.830372	-3.258176
C	-3.198073	-2.211800	-1.971801
C	-3.140288	-1.170536	-3.067168
H	-3.574613	-0.256523	-2.656518
C	-1.694977	-0.856328	-3.445668
H	-1.166652	-0.438097	-2.584426
H	-1.654643	-0.116727	-4.250016
H	-1.166502	-1.752274	-3.786687
C	-3.955812	-1.582443	-4.288908
H	-3.530885	-2.459789	-4.784639
H	-3.973633	-0.770537	-5.020606
H	-4.986856	-1.818212	-4.015796
C	-3.947273	-2.438263	1.780728
H	-4.463312	-1.476659	1.732006
C	-2.675093	-2.222274	2.600024
H	-2.065401	-3.132050	2.630799
H	-2.919367	-1.948398	3.630269
H	-2.084148	-1.406806	2.172200
C	-4.875201	-3.436968	2.462365
H	-5.769201	-3.624425	1.863158
H	-5.191688	-3.049409	3.433949
H	-4.383109	-4.396818	2.642707
C	-4.886210	0.152496	-0.195967
H	-5.828090	-0.340145	-0.386968
C	-4.660739	1.427804	0.183939
H	-5.378848	2.212276	0.373363
C	-2.693664	2.839910	0.681273
C	-2.608891	3.873978	-0.266676
C	-1.935579	5.043379	0.081908
H	-1.857813	5.851222	-0.636300
C	-1.352230	5.186262	1.330182
H	-0.826842	6.101008	1.582393
C	-1.441824	4.159089	2.257862
H	-0.981081	4.280033	3.231163
C	-2.114356	2.976835	1.958865
C	-2.217320	1.849471	2.962241
H	-2.087336	0.927290	2.383912
C	-3.609441	1.805990	3.591106
H	-3.811372	2.724117	4.151328
H	-3.686331	0.962692	4.283517
H	-4.381978	1.688092	2.830201
C	-1.142951	1.880538	4.039167
H	-0.136813	1.931615	3.614720
H	-1.200888	0.970037	4.640473
H	-1.265418	2.727046	4.721725
C	-3.149674	3.674162	-1.667472
H	-4.013389	3.009539	-1.593106
C	-2.110043	2.955552	-2.529818
H	-1.195831	3.553951	-2.614513
H	-2.492818	2.788143	-3.540482
H	-1.866699	1.979043	-2.099441
C	-3.609760	4.963942	-2.333566

H	-4.313479	5.513670	-1.703985
H	-4.107190	4.735487	-3.279235
H	-2.771759	5.627782	-2.564423
B	-2.528925	0.388543	0.010390
K	0.310245	1.854371	-0.041114
N	3.700540	0.498117	-0.318824
N	3.323631	-1.614823	0.280095
C	3.561992	1.863297	-0.652142
C	3.220469	2.219789	-1.966831
C	2.914600	3.552891	-2.237160
H	2.630553	3.840896	-3.244054
C	2.973532	4.515481	-1.242832
H	2.729065	5.547688	-1.469012
C	3.361178	4.158955	0.040738
H	3.420379	4.920925	0.809666
C	3.667080	2.837836	0.356826
C	4.019690	2.439817	1.774678
H	4.586532	1.507739	1.715649
C	2.750921	2.142457	2.574001
H	2.202601	1.313417	2.115025
H	2.995958	1.851906	3.599508
H	2.104475	3.025990	2.622697
C	4.885362	3.471401	2.487556
H	4.337711	4.395806	2.691474
H	5.218400	3.076422	3.450527
H	5.770379	3.726513	1.900008
C	3.150206	1.184724	-3.067210
H	3.558732	0.259083	-2.656360
C	1.703611	0.904935	-3.466144
H	1.196238	1.814811	-3.802164
H	1.658020	0.175894	-4.279852
H	1.158522	0.486921	-2.615485
C	3.990779	1.586957	-4.275643
H	5.024144	1.793238	-3.987826
H	3.996555	0.782178	-5.015451
H	3.594544	2.480407	-4.766333
C	4.927214	-0.141418	-0.168517
H	5.871517	0.356397	-0.333144
C	4.698791	-1.422393	0.191891
H	5.414657	-2.204936	0.396352
C	2.722775	-2.840744	0.636579
C	2.212475	-3.003486	1.935032
C	1.479687	-4.154326	2.223586
H	1.066686	-4.287285	3.218347
C	1.276255	-5.131001	1.263149
H	0.701944	-6.019376	1.502744
C	1.820263	-4.977417	-0.004472
H	1.667548	-5.753931	-0.745255
C	2.551187	-3.840400	-0.338706
C	3.074447	-3.633601	-1.744592
H	3.943770	-2.975956	-1.670689
C	3.518717	-4.925163	-2.418834
H	2.673709	-5.582809	-2.641486
H	4.007420	-4.698437	-3.369514
H	4.224939	-5.480824	-1.797448
C	2.033579	-2.902961	-2.594054
H	1.808149	-1.923575	-2.159512
H	2.406380	-2.739147	-3.609011
H	1.111319	-3.490462	-2.669310
C	2.433225	-1.959592	3.006467
H	3.058707	-1.181175	2.565724
C	1.122466	-1.304822	3.434444
H	0.407721	-2.040361	3.815253
H	1.299373	-0.571991	4.226147
H	0.663411	-0.776106	2.594099
C	3.173489	-2.548892	4.204520
H	4.116811	-3.006375	3.897868
H	3.395637	-1.766485	4.935219
H	2.578801	-3.314849	4.710850
B	2.564408	-0.395014	-0.040072

3'

84

C	-3.12757	3.04055	1.19891
C	-2.46265	3.22559	-0.02171
C	-3.11176	2.98665	-1.24153
C	-4.42559	2.52303	-1.22047
C	-5.08679	2.31416	-0.01875
C	-4.44104	2.57574	1.18109
N	-1.09908	3.58689	-0.02076
B	0.00966	2.62672	0.01676
N	1.12567	3.57875	-0.01122
C	0.69301	4.90057	-0.05968
C	-0.65633	4.90551	-0.06570
C	-2.37580	3.18726	-2.52920
C	-2.40950	3.30084	2.48598
C	2.48649	3.20752	0.00312
C	3.13809	3.02444	1.23119
C	4.44832	2.55023	1.22881
C	5.10375	2.27717	0.03680
C	4.45578	2.48395	-1.17247
C	3.14569	2.95709	-1.20897
C	2.40922	3.29666	2.50969
C	2.42410	3.15663	-2.50492
K	1.84751	-0.00699	0.07836
B	-0.00955	-2.62709	0.02893
N	1.09915	-3.58680	-0.01908
C	2.46266	-3.22535	-0.02358
C	3.13350	-3.05057	1.19531
C	4.44699	-2.58590	1.17503

C	5.08694	-2.31437	-0.02571
C	4.41982	-2.51295	-1.22590
C	3.10581	-2.97615	-1.24450
C	2.42156	-3.32143	2.48362
C	2.36362	-3.16579	-2.53023
C	0.65641	-4.90525	-0.06914
C	-0.69289	-4.90059	-0.05750
N	-1.12556	-3.57920	0.00157
C	-2.48635	-3.20785	0.01386
C	-3.14957	-2.97768	-1.20007
C	-4.45967	-2.50430	-1.16726
C	-5.10353	-2.27692	0.04048
C	-4.44403	-2.52970	1.23474
C	-3.13393	-3.00422	1.24084
C	-2.43159	-3.19698	-2.49482
C	-2.40159	-3.25670	2.52141
K	-1.84817	0.00680	0.04715
H	-4.97725	-2.31219	-2.10175
H	-6.12134	-1.90289	0.05103
H	-4.95006	-2.35892	2.17965
H	-1.37343	-5.73874	-0.08338
H	1.33061	-5.74809	-0.10638
H	4.97099	-2.43727	2.11388
H	6.10763	-1.94814	-0.02615
H	4.92220	-2.30645	-2.16560
H	4.95740	2.39517	2.17480
H	6.12161	1.90337	0.05024
H	4.97036	2.27631	-2.10529
H	1.37362	5.73870	-0.08467
H	-1.33053	5.74860	-0.09691
H	-4.93255	2.32447	-2.15943
H	-6.10738	1.94767	-0.01727
H	-4.96043	2.41908	2.12119
H	-2.98799	2.88923	-3.38175
H	-2.08453	4.23276	-2.65798
H	-1.44563	2.61074	-2.52911
H	-3.03573	3.04768	3.34285
H	-1.48214	2.72141	2.52808
H	-2.11542	4.35035	2.56711
H	3.04250	2.84973	-3.34979
H	1.48961	2.58718	-2.51129
H	2.14216	4.20369	-2.64168
H	3.02381	3.04043	3.37403
H	2.12489	4.34936	2.58412
H	1.47569	2.72671	2.54399
H	-3.01656	-2.99383	3.38350
H	-2.11097	-4.30679	2.60745
H	-1.47136	-2.68090	2.54750
H	-3.04957	-2.89622	-3.34220
H	-1.49371	-2.63332	-2.50952
H	-2.15620	-4.24719	-2.62033
H	2.97250	-2.86261	-3.38332
H	2.06958	-4.20971	-2.66531
H	1.43471	-2.58728	-2.52154
H	3.05122	-3.07366	3.33955
H	1.49343	-2.74390	2.53420
H	2.12947	-4.37197	2.55843

I'

68

C	3.03990	-1.56576	1.31574
C	2.46430	-1.52144	0.04028
C	3.21619	-1.13372	-1.07522
C	4.55189	-0.78358	-0.89652
C	5.13181	-0.81729	0.36239
C	4.37743	-1.20647	1.45897
N	1.10336	-1.85315	-0.12180
B	-0.03620	-0.95860	0.10346
N	-1.11931	-1.85584	-0.30330
C	-0.64331	-3.09974	-0.70337
C	0.70154	-3.09621	-0.59999
C	2.57152	-1.07615	-2.42504
C	2.21829	-1.98430	2.49416
C	-2.49758	-1.55386	-0.28747
C	-3.25704	-1.86041	0.84777
C	-4.60859	-1.52705	0.85845
C	-5.19516	-0.90437	-0.23278
C	-4.43196	-0.60699	-1.35146
C	-3.07782	-0.92606	-1.39558
C	-2.60236	-2.49929	2.03186
C	-2.23702	-0.58821	-2.58673
H	-5.20335	-1.75581	1.73708
H	-6.24956	-0.65077	-0.21139
H	-4.88936	-0.11808	-2.20598
H	-1.29716	-3.89612	-1.02662
H	1.40120	-3.88847	-0.82122
H	5.14020	-0.48029	-1.75727
H	6.17405	-0.54461	0.48874
H	4.83060	-1.23807	2.44475
H	3.23738	-0.61914	-3.15949
H	2.30127	-2.07353	-2.78139
H	1.63734	-0.50907	-2.37620
H	2.80793	-1.95559	3.41198
H	1.34617	-1.33342	2.60192
H	1.82747	-2.99617	2.35930
H	-2.82360	-0.06756	-3.34580
H	-1.39156	0.03810	-2.28656
H	-1.80529	-1.48706	-3.03448
H	-3.30932	-2.61501	2.85488
H	-2.19638	-3.48234	1.77988

H	-1.75464	-1.89424	2.36591
Li	-0.06563	1.15706	0.83833
C	-1.55932	3.71938	1.14894
C	-3.26676	2.68049	-0.21319
C	-2.87744	1.87452	1.01784
H	-0.52644	4.04733	1.03665
H	-1.98228	4.17813	2.05131
H	-4.34223	2.85274	-0.26155
H	-2.97767	2.13525	-1.11250
H	-3.52368	2.10643	1.87386
H	-2.85706	0.79783	0.86110
C	2.88615	2.59527	-1.39575
C	2.70438	2.19580	0.91541
C	3.61900	2.86834	-0.08642
H	3.11315	1.58577	-1.74055
H	3.14093	3.29870	-2.18940
H	2.76251	2.61265	1.92222
H	2.88863	1.11821	0.95648
H	3.68419	3.94244	0.10919
H	4.62371	2.44532	-0.06782
O	1.37423	2.43277	0.42414
O	-1.54637	2.30499	1.31424
C	-2.44978	3.98090	-0.07063
H	-1.84956	4.16354	-0.96346
H	-3.08223	4.85616	0.08538
C	1.42248	2.69484	-0.98895
H	0.79204	1.96063	-1.50063
H	1.01484	3.69702	-1.15836

3' triplet state

84

C	2.53650	3.55540	-0.24836
C	1.74815	2.92476	0.72465
C	2.32604	2.22724	1.79229
C	3.71384	2.15487	1.86069
C	4.50859	2.75543	0.89702
C	3.92253	3.44849	-0.15061
N	0.33933	2.98605	0.62133
B	-0.56721	1.96364	0.13704
N	-1.83674	2.66113	0.16054
C	-1.66373	3.96546	0.61933
C	-0.35964	4.16190	0.89051
C	1.45748	1.57803	2.82233
C	1.90111	4.30480	-1.38038
C	-3.09991	2.18087	-0.26147
C	-3.48863	2.36045	-1.59573
C	-4.69859	1.80600	-2.01017
C	-5.51075	1.12341	-1.11579
C	-5.13087	0.99644	0.21134
C	-3.92632	1.53058	0.66144
C	-2.60213	3.09389	-2.55264
C	-3.50740	1.42251	2.09313
K	-1.87368	-0.54772	-1.58269
B	0.56737	-1.96386	0.13723
N	-0.33921	-2.98610	0.62184
C	-1.74804	-2.92465	0.72489
C	-2.53627	-3.55522	-0.24827
C	-3.92229	-3.44795	-0.15096
C	-4.50848	-2.75457	0.89640
C	-3.71386	-2.15410	1.86022
C	-2.32606	-2.22684	1.79226
C	-1.90068	-4.30493	-1.37999
C	-1.45764	-1.57766	2.82243
C	0.35972	-4.16182	0.89163
C	1.66386	-3.96546	0.62059
N	1.83691	-2.66132	0.16131
C	3.09995	-2.18119	-0.26124
C	3.92653	-1.53042	0.66118
C	5.13081	-0.99615	0.21052
C	5.51030	-1.12350	-1.11669
C	4.69801	-1.80660	-2.01057
C	3.48829	-2.36115	-1.59555
C	3.50796	-1.42184	2.09294
C	2.60163	-3.09507	-2.55195
K	1.87352	0.54731	-1.58350
H	5.77351	-0.47460	0.91147
H	6.44715	-0.69560	-1.45589
H	5.00582	-1.92383	-3.04423
H	2.49211	-4.64928	0.72791
H	-0.13042	-5.04375	1.27453
H	-4.54300	-3.92106	-0.90486
H	-5.58841	-2.68186	0.96060
H	-4.17313	-1.62153	2.68576
H	-5.00671	1.92294	-3.04378
H	-6.44781	0.69562	-1.45453
H	-5.77347	0.47528	0.91267
H	-2.49199	4.64934	0.72617
H	0.13046	5.04397	1.27314
H	4.17299	1.62252	2.68643
H	5.58852	2.68303	0.96157
H	4.54334	3.92165	-0.90439
H	2.05670	1.16995	3.63721
H	0.73884	2.29031	3.23439
H	0.87264	0.77095	2.37013
H	2.62738	4.50507	-2.16927
H	1.06096	3.74913	-1.80747
H	1.49365	5.26239	-1.04445
H	-4.25497	0.88594	2.67775
H	-2.55006	0.90532	2.18066
H	-3.36781	2.41247	2.53482

H	-3.09077	3.21468	-3.51990
H	-2.33264	4.07984	-2.16720
H	-1.65890	2.55683	-2.71558
H	3.09016	-3.21646	-3.51919
H	2.33208	-4.08076	-2.16590
H	1.65846	-2.55799	-2.71511
H	4.25615	-0.88590	2.67736
H	2.55116	-0.90368	2.18057
H	3.36746	-2.41160	2.53476
H	-2.05696	-1.16986	3.63739
H	-0.73881	-2.28983	3.23431
H	-0.87300	-0.77034	2.37041
H	-2.62687	-4.50560	-2.16885
H	-1.06059	-3.74926	-1.80721
H	-1.49309	-5.26231	-1.04367

5. References for supporting information

s1. D. C. Bradley, M. B. Hursthouse, A. A. Ibrahim, K. M. A. Malik, M. Motevalli, R. Möseler, H. Powell, J. D. Runnacles and A. C. Sullivan, *Polyhedron*, 1990, **9**, 2959-2964.

s2. Y. Segawa, M. Yamashita and K. Nozaki, *Science*, 2006, **314**, 113-115; Y. Segawa, Y. Suzuki, M. Yamashita and K. Nozaki, *J. Am. Chem. Soc.*, 2008, **130**, 16069-16079.

s3. Gaussian 16, Revision C.01, M. J. Frisch, G. W. Trucks, H. B. Schlegel, G. E. Scuseria, M. A. Robb, J. R. Cheeseman, G. Scalmani, V. Barone, G. A. Petersson, H. Nakatsuji, X. Li, M. Caricato, A. V. Marenich, J. Bloino, B. G. Janesko, R. Gomperts, B. Mennucci, H. P. Hratchian, J. V. Ortiz, A. F. Izmaylov, J. L. Sonnenberg, D. Williams-Young, F. Ding, F. Lipparini, F. Egidi, J. Goings, B. Peng, A. Petrone, T. Henderson, D. Ranasinghe, V. G. Zakrzewski, J. Gao, N. Rega, G. Zheng, W. Liang, M. Hada, M. Ehara, K. Toyota, R. Fukuda, J. Hasegawa, M. Ishida, T. Nakajima, Y. Honda, O. Kitao, H. Nakai, T. Vreven, K. Throssell, J. A. Montgomery, Jr., J. E. Peralta, F. Ogliaro, M. J. Bearpark, J. J. Heyd, E. N. Brothers, K. N. Kudin, V. N. Staroverov, T. A. Keith, R. Kobayashi, J. Normand, K. Raghavachari, A. P. Rendell, J. C. Burant, S. S. Iyengar, J. Tomasi, M. Cossi, J. M. Millam, M. Klene, C. Adamo, R. Cammi, J. W. Ochterski, R. L. Martin, K. Morokuma, O. Farkas, J. B. Foresman, and D. J. Fox, Gaussian, Inc., Wallingford CT, 2019.

s4. (a) J. P. Perdew, K. Burke, and M. Ernzerhof, *Phys. Rev. Lett.*, **1996**, *77*, 3865-3868, b) J. P. Perdew, K. Burke, and M. Ernzerhof, *Phys. Rev. Lett.*, **1997**, *78*, 1396, c) C. Adamo and V. Barone, *J. Chem. Phys.*, **1999**, *110*, 6158-6169.

s5. (a) A. Schaefer, H. Horn, and R. Ahlrichs, *J. Chem. Phys.*, **1992**, *97*, 2571-2577; (b) A. Schaefer, C. Huber, and R. Ahlrichs, *J. Chem. Phys.*, **1994**, *100*, 5829-5835.

s6. S. Grimme, S. Ehrlich and L. Goerigk, *J. Comp. Chem.*, **2011**, *32*, 1456-1465.

s7. AIMAll (Version 19.10.12), Todd A. Keith, TK Gristmill Software, Overland Park KS, USA, 2019 (aim.tkgristmill.com)
